# Accelerated discovery of superoxide-dismutase nanozymes via high-throughput computational screening

**DOI:** 10.1038/s41467-021-27194-8

**Published:** 2021-11-25

**Authors:** Zhenzhen Wang, Jiangjiexing Wu, Jia-Jia Zheng, Xiaomei Shen, Liang Yan, Hui Wei, Xingfa Gao, Yuliang Zhao

**Affiliations:** 1 Laboratory of Theoretical and Computational Nanoscience, National Center for Nanoscience and Technology of China, Beijing, 100190 China; 2 Department of Biomedical Engineering, College of Engineering and Applied Sciences, Nanjing National Laboratory of Microstructures, Jiangsu Key Laboratory of Artificial Functional Materials, Chemistry and Biomedicine Innovation Center (ChemBIC), Nanjing University, Nanjing, Jiangsu 210023 China; 3 Key Laboratory of Functional Small Organic Molecule, College of Chemistry and Chemical Engineering, Jiangxi Normal University, Nanchang, 330022 China; 4 CAS Key Laboratory for Biomedical Effects of Nanomaterials and Nanosafety, Institute of High Energy Physics and National Center for Nanoscience and Technology, Chinese Academy of Sciences, Beijing, 100049 P. R. China

**Keywords:** Heterogeneous catalysis, Nanoparticles, Density functional theory, Computational chemistry

## Abstract

The activity of nanomaterials (NMs) in catalytically scavenging superoxide anions mimics that of superoxide dismutase (SOD). Although dozens of NMs have been demonstrated to possess such activity, the underlying principles are unclear, hindering the discovery of NMs as the novel SOD mimics. In this work, we use density functional theory calculations to study the thermodynamics and kinetics of the catalytic processes, and we develop two principles, namely, an energy level principle and an adsorption energy principle, for the activity. The first principle quantitatively describes the role of the intermediate frontier molecular orbital in transferring electrons for catalysis. The second one quantitatively describes the competition between the desired catalytic reaction and undesired side reactions. The ability of the principles to predict the SOD-like activities of metal-organic frameworks were verified by experiments. Both principles can be easily implemented in computer programs to computationally screen NMs with the intrinsic SOD-like activity.

## Abstract (web summary)

A general predicting theory for superoxide-dismutase mimicking nanomaterials is currently lacking. The present manuscript reports a density functional theory study on the superoxides dismutase-like activity of nanomaterials based on their electronic band structures and surface adsorption energies.

## Introduction

**Fig. 1:**
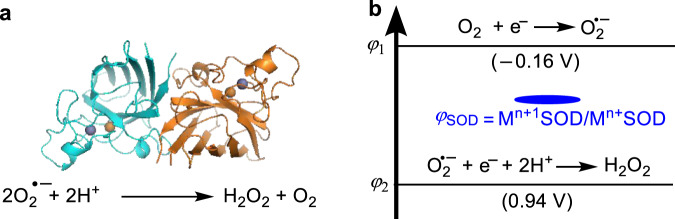
Metalloenzyme catalytically scavenging O_2_^•−^. **a** The superoxide dismutase (SOD) that scavenges O_2_^•−^ by catalyzing its dismutation reaction. **b** The reduction potential model predicting the catalytic activity of SOD. In **b**, *φ*_1_ and *φ*_2_ are the reduction potentials of O_2_/O_2_^−^ and O_2_^−^, H^+^/H_2_O_2_, respectively, and their values at pH = 7 are given in the parentheses.

The principles governing the activity of nanomaterials (NMs) in the catalytically scavenging superoxide anion (O_2_^•−^) underlie many biological effects of the materials, which are of great importance in both basic chemistry and therapeutic applications of the materials. O_2_^•−^ is an unavoidable byproduct of oxygen metabolism. An excess amount of O_2_^•−^ triggers many severe diseases, such as heart disease, cardiovascular disease, cancer, diabetes, Alzheimer’s disease, and Parkinson’s disease^1^. To protect cells from O_2_^•−^, biosystems have evolved a specific family of metalloenzyme called superoxide dismutases (SOD), which catalyze the dismutation of O_2_^•−^ to form biologically less harmful species, hydrogen peroxide (H_2_O_2_) and O_2_ (Fig. 1a). Intriguingly, recent studies have demonstrated that some inorganic NMs possess the activity of scavenging O_2_^•−^, which mimics SOD (Table 1). Compared to SOD, NMs are usually more stable and less expensive. Moreover, such enzyme-like NMs (collectively called nanozymes)^2–5^ can simultaneously possess unique electronic and magnetic properties, which are not exhibited by SOD. Therefore, NMs with SOD-like catalytic activity has shown the potential to develop novel therapeutic strategies^5^ in the area of radiation protection^6^ and the treatment of diseases such as vascular calcification^7^, immunodeficiencies^8^, and neurodegenerative diseases^8^.

**Table 1:** Nanomaterials catalytically scavenging O_2_^•−^ and having atomistic-level structural information for the computational investigation.

Material	Type	Exposed facets	Ref.
sp^2^-carbons	Carbon		^9–14^
Au	Metal		^15^
Pd	Metal	(111), (100)	^16^
Pt	Metal		^17^
MnO			^18^
MnO_2_			^19,20^
Mn_3_O_4_	Spinel	(211), (103), (101)	^21–23^
Co_3_O_4_	Inverse spinel	(220), (311), (400)	^24^
NiO		(111), (200)	^25^
CeO_2_	Fluorite	(111), (200)	^26^
MoS_2_	TMDC		^27^
V_2_C	MXene		^28^
Nb_2_C	MXene		^29^

However, the activities of these NMs in catalytically scavenging O_2_^•−^ are much weaker than those of SODs, hindering their widespread applications^30^. Lacking a general prediction theory, the rational design of NMs with sufficiently high activities along with other desired properties is particularly challenging. Dugan and coworkers suggested a two-step mechanism for water-soluble C_60_ fullerene catalytically scavenging O_2_^•−^. The catalyst is first reduced by accepting an electron from O_2_^•−^, followed by transferring the electron to another O_2_^•−^; these two steps generate O_2_ and H_2_O_2_, respectively, in the presence of protons in the solution^14^. This mechanism has been verified by later studies on the SOD-like properties of other C_60_ derivatives^9,31^, sp^2^-carbon nanoparticles^10,12,13^, and perylene diimide^11^. Self and coworkers experimentally demonstrated that the SOD-like activity of nanoceria increases with the ratio of Ce^3+^ to Ce^4+^ in the material^26^. Using density functional theory (DFT) calculations, we computationally studied the rearrangements of HO_2_^•^ radicals on the surfaces of nanoceria^32^ and noble metals^33^ in the gas phase, which suggested that the catalyst surfaces influence the catalytic activity by tuning the kinetic stability of the intermediate structures involved in the rearrangements^32,33^. Although these results have provided insights into the catalytic mechanisms and have revealed structure-activity relationships for some specific types of NMs, the general principles determining the activity are still elusive. On the other hand, previous studies have established a powerful principle, which is shown in Fig. 1b, for the activity of SOD^34^.

In this work, by learning from the principle of SOD and considering the differences between the NMs and SOD, we develop and verify a new energy level principle and adsorption energy principle for the SOD-like activity of NMs on the basis of their electronic band structures and surface adsorption energies, respectively. The energy level principle reveals the critical role of the intermediate frontier molecular orbital (iFMO), which is defined as the FMO of the NM with energy located in between *φ*_1_ and *φ*_2_, where *φ*_1_ and *φ*_2_ are the potentials of the half-reactions of O_2_^•−^ dismutation, in transferring electrons for catalysis. The adsorption energy principle quantitatively describes the competition between the target catalytic reaction and possible side reactions for O_2_^•−^ on the catalysts. Therefore, the combination of both principles provides not only systematic and in-depth insight into the mechanisms of the SOD-like properties of NMs but also a general guide for the computational design and screening of SOD-like NMs.

## Results and discussion

### Energy level principle

We first developed a theoretical model to predict the activity of NMs catalyzing the dismutation of O_2_^•−^. In nature, SOD is the enzyme that specifically catalyzes this reaction, and previous studies have achieved a thermodynamics-based model (Fig. 1b)^34^. According to this model, the reduction potential order *φ*_1_ < *φ*_SOD_ < *φ*_2_ is the sufficient and necessary condition for the activity of SOD. This condition ensures that both half-reactions of the dismutation are thermodynamically spontaneous and that the maximized activity is reached when *φ*_SOD_ = (*φ*_1_ + *φ*_2_)/2^34^. Because all reaction steps involved in SOD catalysis have low energy barriers^34^ and the catalyzes are under thermodynamic control, such a purely thermodynamics-based theoretical principle of SOD has stood the test of time^34^.

To develop a prediction model for NMs by learning from SOD, the following two differences between the NM and SOD must be considered. First, the electron energy levels responsible for electron transfer in catalysis are different. In SODs, the level is *φ*_SOD_, i.e., the potential of M^n+1^SOD/M^n^SOD. In contrast, those of NMs are FMOs, which include the conduction band minimum (CBM), valence band maximum (VBM), or mid-gap impurity level (MGIL). For bandgap NMs, the MGILs are associated with structural imperfections; for metal NMs, the MGILs are Fermi energy levels (*E*_F_). Second, the possibilities of side reactions in the two cases are different. Because of its protein scaffolds, SOD catalyzes the dismutation of O_2_^•−^ with high specificity. By contrast, side reactions may compete with the target catalytic reaction occurring on the NMs. These two differences suggest the necessity of developing new principles for the SOD-like activity of NMs.

**Fig. 2:**
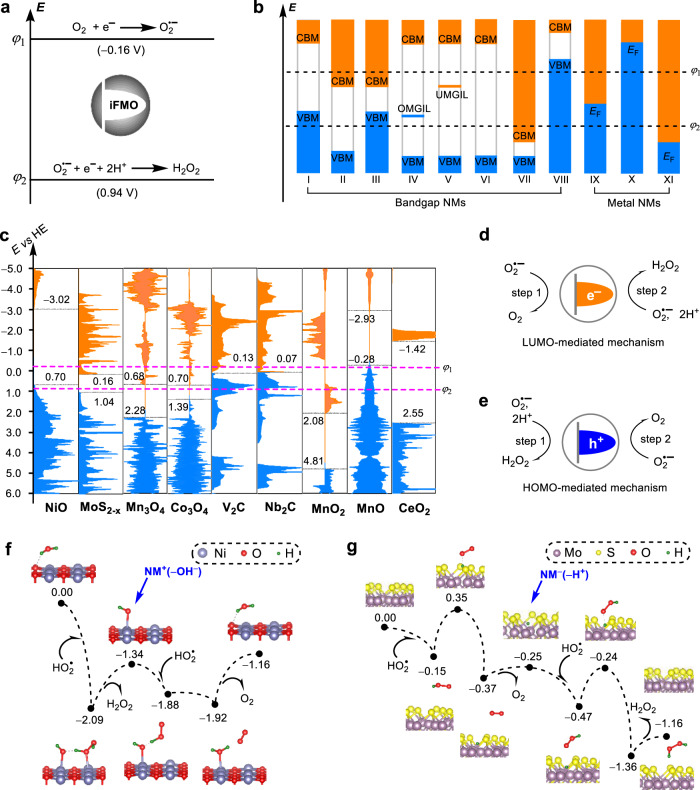
The energy level principle for NMs to catalytically scavenge O_2_^•−^. **a** The model for nanomaterials, where intermediate frontier molecular orbital (iFMO) means the FMO with energy located in the range (*φ*_1_, *φ*_2_). **b** Nanomaterials having different types of FMOs with respect to the potentials *φ*_1_ and *φ*_2_. **c** Calculated electronic density of states with the energies of FMOs marked. (**d**, **e**) Diagrams for the LUMO- and HOMO-mediated catalytic mechanisms. (**f**, **g**) Reaction energy profiles corresponding to the superoxide dismutase-like catalyzes of NiO(100) and MoS_2−x_, respectively; the intermediates with the ionic nature are labeled. In (**f**, **g**) some water molecules as explicit solvents and the kinetically unfavorable pathways are not shown for clarity; the complete energy profiles are given in Supplementary Fig. 1. In **b**–**d**, and **e**, the blue and orange colors represent the occupied and unoccupied molecular orbitals, respectively.

Because FMOs are responsible for electron transfer in the case of NMs, we hereby adjust *φ*_SOD_ in the model of SODs to the FMO energy (*E*_FMO_) to fit with the NMs. A similar idea of using FMO energies as activity descriptors has been proposed for the oxidative dehydrogenation reactions catalyzed by CeO_2_-based catalysts^35,36^. Therefore, we obtain the energy level principle for NMs (Fig. 2a). According to this principle, we propose Eq. (1)

1 $${\varphi }_{1} < \, {E}_{{{{{{\rm{FMO}}}}}}} \, < \, {\varphi }_{2}$$

to be the first criterion for screening NMs as O_2_^•−^ scavenging catalysts. Thereafter, we will denote the FMO satisfying Eq. (1) as iFMO. Similar to the case of SOD, when *E*_iFMO_ = (*φ*_1_ + *φ*_2_)/2, the maximized catalytic efficiency of the NM will be anticipated when the other factors influencing the activity are the same. With this principle, it is easy to predict whether a NM is a potential SOD mimic. Figure 2b illustrates XI types of electronic band structures of NMs: I − VIII are characteristics of bandgap NMs, and IX − XI are those of metal NMs. Among them, I, II, IV, V, and IX have a VBM, a CBM, an occupied MGIL (OMGIL), an unoccupied MGIL (UMGIL), and an *E*_F_ in the range (*φ*_1_, *φ*_2_), respectively; III has both a VBM and a CBM in this range. Accordingly, these NMs have iFMOs and thus are potential catalysts. In contrast, VI, VII, VIII, X, and XI have no iFMOs and thus are not the potential catalysts. Because the FMO energies of NMs can be measured by experiments or calculated by DFT, this energy level principle could be easily implemented for screening NMs toward O_2_^•−^ scavenging catalysts.

To verify the energy level principle, we have calculated the electronic density of states (DOSs) for all materials listed in Table 1, whose SOD-like catalytic activities have been experimentally confirmed before. For these calculations, graphene has been used as the model of sp^2^ carbons. Reportedly, MoS_2_ contains sulfur vacancies^37^. Therefore, MoS_2-x_ with a reduced number of sulfur has been used for the calculation (for computational method, see Supplementary Fig. 2, Supplementary Table 1, and Supplementary Method). The calculated results are shown in Fig. 2c and Supplementary Fig. 3a. NiO, MoS_2-x_, Mn_3_O_4_, Co_3_O_4_, V_2_C, Nb_2_C, Au, and Pd indeed have FMOs located in the energy window (−0.16 V, 0.94 V). The Fermi level of graphene is −0.19 V, which is close to −0.16 V. These results excellently support the energy level principle. The CBM of MnO_2_ has energy more positive than *φ*_2_; therefore, MnO_2_ will probably oxidize O_2_^•−^ to form O_2_, H_2_O, and MnO_2-x_ in the presence of H^+^ rather than catalyze the disproportionation of O_2_^•−^. In contrast, the VBM of MnO has energy more negative than *φ*_1_; therefore, MnO will probably reduce O_2_^•−^ to form H_2_O_2_ and MnO_1+x_ in the presence of H^+^. We reason that the SOD-like activities experimentally found for them are actually originated from the modified structures MnO_2-x_, and MnO_1+x_, respectively. Similarly, the SOD-like activity of CeO_2_ is originated from the modified structure CeO_2-x_^26,32^. Pt does not have any iFMO, which can be ascribed to that Pt particles practically expose irregular surfaces unlike the Pt(111) used for the calculation. These results suggest the potential power of the principle to investigate the in-situ active structures for the catalysts.

Besides the support by computation, such an energy level principle has also been supported by previous experimental results on the redox potentials of the materials. For example, Tsai et al. reported the SOD-like activity of sp^2^ carbon NMs, including single-walled carbon nanotubes and nanographene^11^. These researchers found that the reduction potential of nanographene was 0.40 V, which was exactly located in the range from −0.16 V to 0.94 V (Fig. 1b), supporting our energy level principle^11^. Recently, Wei *et al*.^28^ have reported the SOD-like activities for V_2_C MXene. The reduction potential of V_2_C has been determined to be −0.11 V^28^, which is in good agreement with our energy level principle.

The energy level principle provides not only the criterion to screen out NMs as potential catalysts but also new insight into the catalysis mechanism. Because the iFMOs of NMs II and V are the unoccupied FMOs (i.e., the lowest unoccupied molecular orbitals, LUMOs), the catalysis follows a LUMO-mediated mechanism (Fig. 2d):

$${{{{{{{\rm{O}}}}}}}_{2}}^{{{{{\bullet }}}}-}+{{{{{\rm{NM}}}}}}={{{{{{\rm{O}}}}}}}_{2}+{{{{{{\rm{NM}}}}}}}^{-}$$

$${{{{{{{\rm{O}}}}}}}_{2}}^{{{{{\bullet }}}}-}+{{{{{{\rm{2H}}}}}}}^{+}+{{{{{{\rm{NM}}}}}}}^{-}={{{{{{\rm{H}}}}}}}_{2}{{{{{{\rm{O}}}}}}}_{2}+{{{{{\rm{NM}}}}}}$$

The NM is first reduced by one O_2_^•−^ and then oxidized back to its original state by another O_2_^•−^, with negatively charged NM^−^ as the intermediate. This CBM-mediated mechanism has been experimentally observed for the SOD-like activity of sp^2^ carbon NMs^11^ having unoccupied iFMOs, which agrees with this prediction. By contrast, the activity of NM-I and NM-IV is mediated by occupied FMOs (i.e., the highest occupied molecular orbitals, HOMOs) and thus follows a HOMO-mediated mechanism (Fig. 2e):

$${{{{{{{\rm{O}}}}}}}_{2}}^{{{{{\bullet }}}}-}+{{{{{{\rm{2H}}}}}}}^{+}+{{{{{\rm{NM}}}}}}={{{{{{\rm{H}}}}}}}_{2}{{{{{{\rm{O}}}}}}}_{2}+{{{{{{\rm{NM}}}}}}}^{+}$$

$${{{{{{{\rm{O}}}}}}}_{2}}^{{{{{\bullet }}}}-}+{{{{{{\rm{NM}}}}}}}^{+}={{{{{{\rm{O}}}}}}}_{2}+{{{{{\rm{NM}}}}}}$$

The NM is first oxidized by one O_2_^•−^ and then reduced back by another with positively charged NM^+^ as the intermediate. Having both occupied and unoccupied FMOs as the iFMOs, the activities of NM-III and NM-IX follow both mechanisms with the overall neutral intermediate. The HOMO-mediated mechanism has not been experimentally reported before and deserves attention in the future.

Using the energy level principle, we now analyze the iFMOs for the NMs in Table 1 to predict the mechanisms of their SOD-like activities, which have been rarely studied at the microscopic level. The results of Fig. 2c and Supplementary Fig. 3a predict the following new knowledge. The iFMO of NiO is its VBM; those of MoS_2−x_, Mn_3_O_4_, and Co_3_O_4_ are their CBMs. Therefore, NiO has an occupied iFMO, following the HOMO-mediated mechanism. MoS_2-x_, Mn_3_O_4_, and Co_3_O_4_ have unoccupied iFMOs, following the LUMO-mediated mechanism. For metals, the Fermi energies with respect to the vacuum energy are known as work function (*W*_f_). Therefore, a *W*_f_-version of the energy level principle has been further derived, which also supports the principle (Supplementary Note 1 and Supplementary Fig. 3b).

To computationally verify the above-predicted mechanisms, we located the key intermediate and transition state structures involved in the dismutation of O_2_^•−^ on the surfaces of NiO(100) and MoS_2-x_ using DFT calculations. As can be seen from Fig. 2f and 2 g, the kinetically favorable pathways for the dismutation of O_2_^•−^ on NiO(100) and MoS_2-x_ indeed follow the HOMO- and LUMO-mediated mechanisms, respectively. On NiO(100), the first protonated O_2_^•−^ (i.e., HO_2_^•^) takes the hydrogen atom from the H_2_O adsorbate to form the H_2_O_2_ molecule, which oxidizes the NM by forming the intermediate NM^+^( − OH^−^). The second HO_2_^•^ transfers its hydrogen to the HO adsorbate to generate the O_2_ molecule, which reduces the NM back to the original state (Fig. 2f). On MoS_2-x_, HO_2_^•^ first passes its hydrogen to the sulfur of MoS_2-x_, reducing the NM by forming the intermediate NM^−^( − H^+^). The second HO_2_^•^ takes the hydrogen away, oxidizing the NM to its original state (Fig. 2g). Therefore, the results of Fig. 2f, g excellently support the predicted mechanisms. The energy profiles in Fig. 2f, g also suggest that the SOD-like catalysts have only very small energy barriers (<0.50 eV), which means that the catalysts are controlled by thermodynamics. This explains why the energy level principle, which only reflects the thermodynamics of the half-reactions, can successfully predict the catalytic activity of NMs.

### Adsorption energy principle

**Fig. 3:**
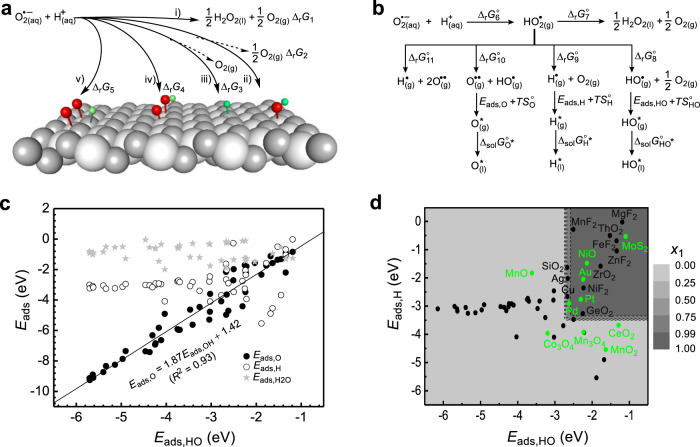
The adsorption energy principle for NMs to catalytically scavenge O_2_^•−^. **a** The dismutation (i) and competitive side reactions (ii−v) of O_2_^•−^ that may occur on the surface of nanomaterials. **b** The Hess cycles for reactions (i−v). **c** The adsorption energies of O^••^, H^•^, and H_2_O with respect to that of HO^•^ on various material surfaces. **d** The contour plot of the partition function *x*_1_ as a function of *E*_ads,HO_ and *E*_ads,H_. In **a**, O and H are shown in red and green, respectively; the other atoms are of the substrate. In **d**, the filled green and black circles represent nanomaterials whose superoxide dismutase-like activities have and have not been experimentally reported.

We now develop a model to describe the competition of the side reactions with the target dismutation of O_2_^•−^ on NMs. In Fig. 3a, the target reaction is illustrated as (i), and the possible side reactions are illustrated as (ii−v). Denoting the Gibbs free energy (*G*) changes in these five reactions as ∆_r_*G*_i_ (i = 1−5), the molar fraction *x*_1_ for O_2_^•−^ to undergo reaction (i) when reaching thermodynamic equilibrium can be expressed with the following partition function:

2 $${x}_{1}=\frac{{e}^{-\tfrac{{\varDelta }_{{{{{{\rm{r}}}}}}}{G}_{1}}{RT}}}{{\sum }_{i=1}^{5}{e}^{-\tfrac{{\varDelta }_{{{{{{\rm{r}}}}}}}{G}_{i}}{RT}}}$$

In Eq. (2), *e*, *R*, and *T* are Euler’s number, the universal gas constant, and temperature, respectively. The target reaction is dominant, meaning that over 50% of O_2_^•−^ undergoes reaction (i). Therefore, we propose *x*_1_ > 0.5 as the second criterion for screening NMs as O_2_^•−^ scavenging catalysts.

To derive a simpler screening criterion from Eq. (2), the mathematical operations described in the Method and Supplementary Method sections were conducted. Briefly, the calculation of *x*_1_ at any pH needs to calculate ∆_r_*G*_i_ (i = 1−5) at the pH. To calculate ∆_r_*G*_i_ (i = 1−5) using the standard thermodynamic data that can be consulted from the chemistry handbooks, the Hess cycles of Fig. 3b have been constructed. The result suggests that *x*_1_ is a function of three variables, *E*_ads,HO_, *E*_ads,O_, and *E*_ads,H_, which represent the energy changes for the adsorptions of HO^•^, O^••^, and H^•^ on the material surfaces, respectively. Because of the linear relationship between *E*_ads,HO_ and *E*_ads,O_ shown in Fig. 3c, *x*_1_ turns to a function of only two variables, *E*_ads,HO_, and *E*_ads,H_. Figure 3d plots the variation in *x*_1_ with *E*_ads,HO_ and *E*_ads,H_. From Fig. 3d, the criterion *x*_1_ > 0.5 becomes

3 $${E}_{{{{{{\rm{ads}}}}}},{{{{{\rm{HO}}}}}}} \; > \; {-}\!2.7\,{{{{{\rm{eV}}}}}}\,{{{{{\rm{and}}}}}}\,{E}_{{{{{{\rm{ads}}}}}},{{{{{\rm{H}}}}}}} \; > \; {-}\!3.4\,{{{{{\rm{eV}}}}}}$$

Therefore, Eq. (3) is the second criterion for screening NMs as O_2_^•−^ scavenging catalysts under the condition that *T* = 298.15 K and pH = 7.

The adsorption energy principle reasonably explains the SOD-like activity for Au^15^, Pd^16^, Pt^17^, NiO^25^, and MoS_2_^27^, which are located in the region with *x*_1_ > 0.5 (Fig. 3d). However, CeO_2_^26^, MnO^18^, MnO_2_^20^, Mn_3_O_4_^22^, and Co_3_O_4_^24^ are beyond the region, although their SOD-like activities have been experimentally demonstrated. Such inconsistency may be ascribed to the that the practical surfaces of these NMs exhibiting activity are different from the slab models used for calculating the *E*_ads,HO_ and *E*_ads,H_. Unfortunately, the exact surface structures of NMs in water are usually difficult to characterize because they may simultaneously expose multiple surfaces^40^, and surface reconstructions may drastically occur in water^41^. We believe that NMs such as CeO_2_, MnO, MnO_2_, Mn_3_O_4_, and Co_3_O_4_ will enter the region *x*_1_ > 0.5 when their exact surface structures are determined and used for calculating the adsorption energies. This point is already supported by the case of CeO_2_. The *E*_ads,H_ of CeO_2_ calculated using the defect-free (111) surface is −3.68 eV. However, practical nanoceria contains oxygen vacancies^38,39^ and thus are more chemically reductive. Therefore, the practical value of *E*_ads,H_ is more positive, pushing CeO_2_ into the region with *x*_1_ > 0.5. These results suggest that Eq. (3) is a simple but powerful criterion that is able to screen our NMs on which the target catalytic reaction is more thermodynamically completive than unwanted side reactions.

### Experimental verification

**Fig. 4:**
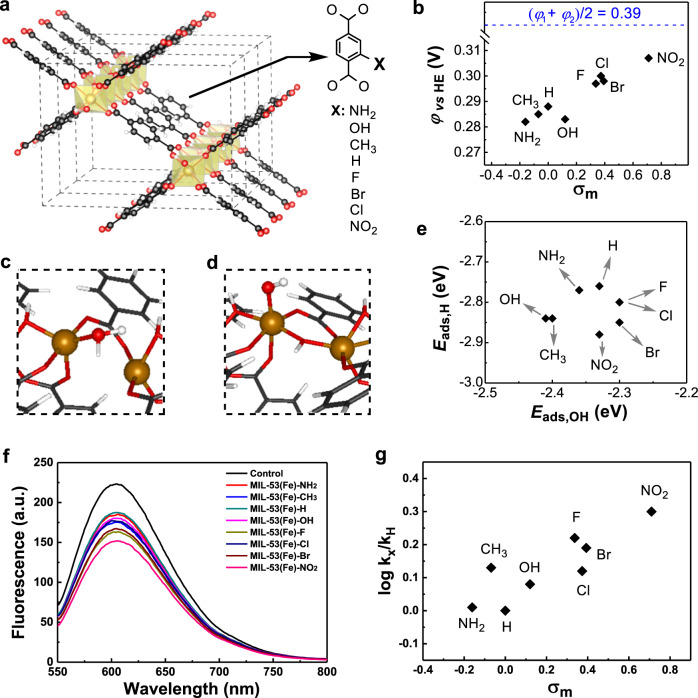
Experimental verification of the screening principles. **a** Structural diagram of MIL-53(Fe)-X. **b** Reduction potentials experimentally determined for MIL-53(Fe)-X with respect to the potential of hydrogen electrode (HE) at pH = 7. **c** Partial structure of MIL-53(Fe)-H with an H adsorbate. **d** Partial structure of MIL-53(Fe)-H with a HO adsorbate. **e** *E*_ads,H_ plotted as a function of *E*_ads,HO_ for MIL-53(Fe)-X. **f** Fluorescent spectra for monitoring the superoxide dismutase-like activities of MIL-53(Fe)-X. **g** Superoxide dismutase-like activities of MIL-53(Fe)-X plotted as a function of Hammett *σ*_m_ values. In **a**, **c**, and **d**, atoms C, O, Fe, and H are shown in black, red, brown, and white, respectively.

To verify the above screening principles, metal-organic framework (MOF) compounds MIL-53(Fe)-X (X = NH_2_, CH_3_, H, HO, F, Cl, Br, and NO_2_) were synthesized and their activities of scavenging O_2_^•−^ were investigated by the combination of experiments and computations. As seen from Fig. 4a, the structures of these MOFs differ from each other in the linker substituents X. The Hammett *σ*_m_ values, which characterize the electron-withdrawing ability of the substituents, increase in the order NH_2_ < CH_3_ < H < HO < F < Cl < Br < NO_2_. To determine whether these MOFs satisfy the energy level criterion (Eq. (1)), their reduction potentials were measured using the cyclic voltammetry technique^42^. Fig. 4b plots the measured reduction potentials as a function of *σ*_m_. The reduction potentials and the *σ*_m_ values have similar variation tendencies, which is in agreement with the knowledge that a stronger electron-withdrawing substituent leads to a high electron affinity of the material^42^. Importantly, the reduction potentials vary from 0.28 V (for X = NH_2_) to 0.31 V (for X = NO_2_), which are exactly located in the range from −0.16 V to 0.94 V. Therefore, all the eight MOFs satisfy the energy level criterion. To determine whether these MOFs satisfy the adsorption energy criterion Eq. (3), the *E*_ads,HO_ and *E*_ads,H_ values at the defective sites of the MOFs were calculated using the DFT method. The most energetically favorable adsorption sites for H^•^ and HO^•^ are the bridge oxygen and iron atoms, respectively (Fig. 4c, d). All the calculated *E*_ads,HO_ values are more negative than −2.3 eV, and those of *E*_ads,H_ more negative than −2.7 eV. Namely, all the eight MOFs also satisfy the adsorption energy level criterion, and thus all the MOFs are predicted to have the activity of catalytically scavenging O_2_^•−^.

The above prediction encouraged us to further investigate the SOD-like activities of MIL-53(Fe)-X MOFs experimentally. The O_2_^•−^ scavenging abilities of MIL-53(Fe)-X MOFs were characterized by monitoring the fluorescent spectra of ethidium, the product of O_2_^•−^ and its specific probe hydroethidine. As shown in Fig. 4f, in the absence of MIL-53(Fe)-X MOFs, the hydroethidine could react with O_2_^•−^ to generate the strongest fluorescence with a peak located around 600 nm. With the introduction of MIL-53(Fe)-X MOFs rather than their corresponding ligands (Supplementary Fig. 4), the fluorescent intensity of ethidium significantly decreased, whereas the one with MIL-53(Fe)-NO_2_ gave the lowest intensity. This result illustrated that the addition of these MIL-53(Fe)-X MOFs could eliminate O_2_^•−^ amount, and thus reducing the fluorescent intensities of ethidium. The SOD-like activities of MIL-53(Fe)-X MOFs were also plotted as a function of *σ*_m_ values (Fig. 4g). And a similar change tendency as the one in Fig. 4b was observed, where MIL-53(Fe)-NH_2_ with the reduction potential farthest and MIL-53(Fe)-NO_2_ with the reduction potential closest to the midpoint of *φ*_1_ and *φ*_2_ (0.39 V) gave the worst and best SOD-like activities. This activity order supported the prediction that NMs with *E*_iFMO_ = (*φ*_1_ + *φ*_2_)/2 have the maximized catalytic efficiency. While for another MIL-47(V)-X (X = H, NH_2_, and Br) MOF, their reduction potentials were even lower than that of MIL-53(Fe)-NH_2_ (Supplementary Fig. 5). And our previous work showed that MOFs including MIL-47(V)-NH_2_, MIL-47(V)-H, and MIL-47(V)-Br indeed exhibited much weaker and even negligible SOD-like activities^43^. Furthermore, DFT calculations suggested that the *E*_ads,HO_ and *E*_ads,H_ of these MIL-47(V) MOFs were about −0.76 eV and more −4.25 eV (Supplementary Method and Supplementary Figs. 6 and 7), respectively, disobeying the adsorption energy criterion Eq. (3). This finding confirmed the negligible SOD-like activities of MIL-47(V)-X (X = H, NH_2_, and Br) MOF, consistent with the guidance of the above principle. The principle was further verified by the different SOD-like activities of ceria particles that were synthesized under the same condition except at different temperatures: 0, 30, 60, and 90 °C. The former two ceria particles had markedly stronger activities than the latter, which exactly agreed with the experimental result that the former had the iFMO but the latter did not (see Supplementary Note 2 and Supplementary Figs. 8‒11). Therefore, the results here not only demonstrated the above screening principles could predict the SOD-like activities of MOFs, but also verified the validity and generality of the principles.

### Computational screening of antioxidants from 2D materials

We now use the above screening principles, Eq. (1) and Eq. (3), to screen NMs with intrinsic SOD-like catalytic activity. Because two-dimensional (2D) materials have large surface areas, which will serve as potential catalytically active sites, we focus our attention on 2D materials. Thygesen and coworkers established the Computational 2D Materials Database (C2DB), which deposits structural, elastic, thermodynamic, electronic, optical, and magnetic properties for a total of 3814 2D materials obtained by DFT calculations^44^. This database provides an excellent platform for the modeling and design of 2D materials without redundant calculations.

**Fig. 5:**
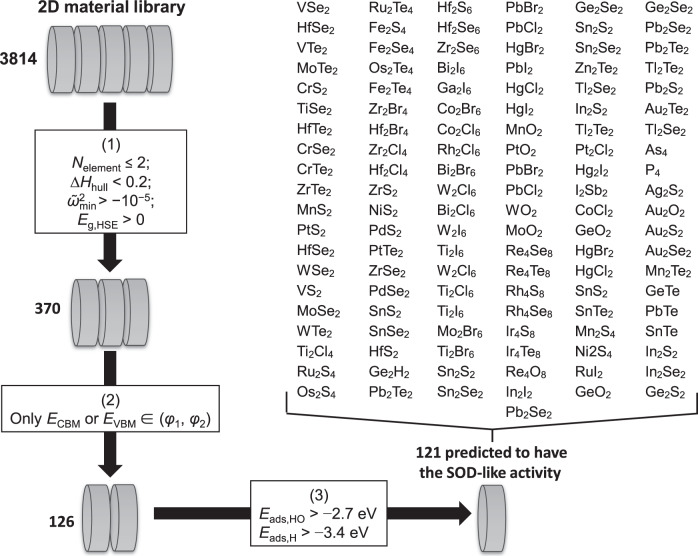
Computational screening of two-dimensional nanomaterials as potential superoxide dismutase mimics. The screening was based on the library containing 3814 materials. Among them, 370 ones, which contained no more than two elements, had considerable thermodynamic and kinetic stabilities, and had an electronic bandgap, passed the first step of screening. 126 ones, which had conduction band minimum or valence band maximum as the only intermediate frontier molecular orbital, passed the second step of screening. 121 ones, which further satisfied the adsorption energy criterion, passed the second step of screening and were predicted to have intrinsic superoxide dismutase-like catalytic activity.

Here, we will screen out stable NMs belonging to type I or II from the 2D material library. We further restrict our screening within the subset composed of no more than two elements because NMs consisting of fewer elements will be more suitable for mechanistic studies in the future. The screening procedure is shown in Fig. 5. A total of 370 materials among the 3814 passed the first-step screening. These materials are all composed of no more than two elements (*N*_element_ ≤ 2), have considerable thermodynamic stability (∆*H*_hull_ < 0.2 eV/atom) and kinetic stability (${\tilde{{{\upomega }}}}_{{{{{{\rm{min}}}}}}}^{2} \, > \, {10}^{-5}{{{{{{\rm{eV}}}}}}/{{\AA }}}^{2}$), and have nonzero electronic bandgaps calculated with the Heyd–Scuseria–Ernzerhof (HSE) method (*E*_g,HSE_ > 0)^44^. A total of 126 of the 370 passed the second-step screening. These materials have only one type of iFMO, either a CBM or a VBM, whose energy is located in the range (*φ*_1_, *φ*_2_). 121 among the 126 passed the third-step screening, which had *E*_ads,HO_ > − 2.7 eV and *E*_ads,H_ > − 3.4 eV. These 121 materials are thus predicted to have intrinsic SOD-like catalytic activities. These results have demonstrated that the screening principles can be easily implemented in computer programs for the high-throughput screening of NMs with intrinsic SOD-like activity. Among these materials, T-phase transition metal dichalcogenides (T-TMDCs, PtS_2_^45^, ZrS_2_^46^, ZrSe_2_^46^, SnS_2_^47^, HfS_2_^48^) and H-TMDCs (MoTe_2_^49^, WSe_2_^50^, WTe_2_^51^, VS_2_^52^) have been experimentally reported. They existed in the form of single layers supported on substrates or a few layers in solutions^53–57^. The SOD-like activities of these nine single-layer TMDC structures have been computationally investigated, and the results match well with the prediction (see Supplementary Table 2, Supplementary Note 3, and Supplementary Figs. 12‒14). Therefore, the screening performed on 2D materials is likely to stimulate the application of these materials as novel SOD mimics provided that the high-yield synthesis of these single layers is achieved.

It’s worth noting that the principles are proposed for ideal material surfaces with explicit atomic structures. However, real NMs usually have inhomogeneous morphologies and ambiguous structural configurations. In addition, because the SOD-like catalysis occurs in aqueous solutions, other molecules present in the solvents may cause irreversible structural modifications to the materials, changing their FMO energies and surface affinities. The different aqueous dispersibility of materials may also influence their practical catalytic performances. This complexity of NMs in real situations should be considered when linking the predicted results to experiments.

In summary, two principles governing the catalytic activity of NMs mimicking that of SOD have been revealed by computationally studying the microscopic thermodynamics and kinetics of the catalytic processes. The energy level principle suggests the critical role of the iFMO in transferring electrons for the catalysis, which can be used to predict whether both half-reactions of the catalytic dismutation of O_2_^•−^ are thermodynamically favorable. This principle suggests the first criterion for screening NMs with the SOD-like activity: the NMs should have at least one iFMO, namely, the FMO with energy located in between (*φ*_1_, *φ*_2_). Because the catalytic mechanism critically depends on whether the iFMO is occupied by electrons or not, this principle determines whether the SOD-like catalysis of NMs follows the HOMO- or the LUMO-mediated mechanism. The adsorption energy principle is based on the thermodynamic partition function and determines whether the target catalytic reaction occurring on the NMs is more thermodynamically competitive than the undesired side reactions. This principle suggests the second criterion for the screening, *E*_ads,HO_ > − 2.7 eV and *E*_ads,H_ > − 3.4 eV at pH 7 and at the level of theory used for the computations. Both principles have received support from the previous results of NMs showing the SOD-like catalytic activity, giving an in-depth explanation of the results. The ability of the principles to predict the SOD-like activities of MOFs was further verified by our experimental results. The screening criteria derived from the principles can be easily implemented into computer programs, and they are used to computationally screen the NMs with the intrinsic SOD-like activity from the database of 2D materials. The results provide a systematic view of the antioxidant property of NMs and the method for the computational design and screening of NMs as novel SOD mimics.

## Methods

### DFT calculations

All NMs were computed using the projector augmented wave (PAW) method^58^ implemented in the Vienna Ab initio Simulation Package (VASP)^59^. The Perdew–Burke–Ernzerhof (PBE)^60^ exchange-correlation functional with the generalized gradient approach (GGA) was applied. The plane-wave energy cutoff was set to 500 eV for fluorites and 400 eV for the other NMs. The GGA + U^61^ corrections using the formalism of Dudarev et al^62^. were also used to accurately describe the distribution of electrons on the NMs surfaces. When calculating the reaction energy profiles for NiO(111) and MoS_2−x_, the water solvent effect was considered by an implicit solvation model using the VASPsol package developed by Kiran Mathew and Richard G. Hennig^63,64^. A vacuum value of 15 Å along the *z*-direction was set to separate the slab with an upward unit cell to avoid inveracious interactions. Gaussian smearing with a width of 0.05 eV was used for the Fermi level. The total energy and force convergences were 10^−5^ eV and 0.02 eV/Å for the local minimum of all potential surfaces. Some small molecules, such as H_2_O, H_2_O_2_, O^••^, HO^•^, HO_2_^•^, and O_2,_ were calculated in a 15 Å cubic lattice using the same parameters and convergence criteria as those for the rutile slabs. For the transition state search, the climbing image nudged elastic band method^65^ was used in conjunction with partial atomic fixation with a convergence of 10^−4^ eV and 0.05 eV/Å. More computational parameters, such as the model of every nanomaterial, the effect value of *U* (*U*_eff_), and the Brillouin zone k-points, are shown in Supplementary Data 1.

The adsorption energy (*E*_ads_) of a molecule on the slab is defined as follows:

$${{E}}_{{\mathrm ads}{,}{\mathrm mol}}={{E}}_{{{{{{\rm{mol}}}}}}@{{{{{\rm{slab}}}}}}}-({{E}}_{{{{{{\rm{slab}}}}}}}+{{E}}_{{{{{{\rm{mol}}}}}}})$$

where *E*_slab_, *E*_mol_, and *E*_mol@slab_ are the total energies for the isolated ceria slab, isolated molecule, and ceria with the adsorbed molecule, respectively. The source data obtained by DFT calculations for Figs. 2–4 are present in Supplementary Data 2.

### Derivation of the adsorption energy criterion

To calculate the partition function *x*_1_ with Eq. (2), the values of ∆_r_*G*_*i*_ (*i* = 1 − 5) should be calculated first. According to the Hess cycles of Fig. 3b, the ∆_r_*G*_*i*_ at any pH can be expressed as follows:

4a $${\varDelta }_{{{{{{\rm{r}}}}}}}{G}_{1}={\varDelta }_{{{{{{\rm{r}}}}}}}{G}_{6}^{^\circ }+0.0592{{{{{\rm{pH}}}}}}+{\varDelta }_{{{{{{\rm{r}}}}}}}{G}_{7}^{^\circ }$$

4b $${\varDelta }_{{{{{{\rm{r}}}}}}}{G}_{2}={\varDelta }_{{{{{{\rm{r}}}}}}}{G}_{6}^{^\circ }+0.0592{{{{{\rm{pH}}}}}}+{\varDelta }_{{{{{{\rm{r}}}}}}}{G}_{8}^{^\circ }+{E}_{{{{{{\rm{ads}}}}}},{{{{{\rm{HO}}}}}}}+T{S}_{{{{{{\rm{HO}}}}}}}^{^\circ }+{\varDelta }_{{{{{{\rm{sol}}}}}},2}{G}_{{{{{{{\rm{HO}}}}}}}^{\ast }}^{^\circ }$$

4c $${\varDelta }_{{{{{{\rm{r}}}}}}}{G}_{3}={\varDelta }_{{{{{{\rm{r}}}}}}}{G}_{6}^{^\circ }+0.0592{{{{{\rm{pH}}}}}}+{\varDelta }_{{{{{{\rm{r}}}}}}}{G}_{9}^{^\circ }+{E}_{{{{{{\rm{ads}}}}}},{{{{{\rm{H}}}}}}}+T{S}_{{{{{{\rm{H}}}}}}}^{^\circ }+{\varDelta }_{{{{{{\rm{sol}}}}}},2}{G}_{{{{{{{\rm{H}}}}}}}^{\ast }}^{^\circ }$$

4d $${\varDelta }_{{{{{{\rm{r}}}}}}}{G}_{4}=\, {\varDelta }_{{{{{{\rm{r}}}}}}}{G}_{6}^{^\circ }+\,0.0592{{{{{\rm{pH}}}}}}+{\varDelta }_{{{{{{\rm{r}}}}}}}{G}_{10}^{^\circ }+{E}_{{{{{{\rm{ads}}}}}},{{{{{\rm{HO}}}}}}}+T{S}_{{{{{{\rm{HO}}}}}}}^{^\circ }\\ +{\varDelta }_{{{{{{\rm{sol}}}}}},2}{G}_{{{{{{{\rm{HO}}}}}}}^{\ast }}^{^\circ }+{E}_{{{{{{\rm{ads}}}}}},{{{{{\rm{O}}}}}}}+T{S}_{{{{{{\rm{O}}}}}}}^{^\circ }+{\varDelta }_{{{{{{\rm{sol}}}}}},2}{G}_{{{{{{{\rm{O}}}}}}}^{\ast }}^{^\circ }$$

4e $${\varDelta }_{{{{{{\rm{r}}}}}}}{G}_{5}=\, {\varDelta }_{{{{{{\rm{r}}}}}}}{G}_{6}^{^\circ }+0.0592{{{{{\rm{pH}}}}}}+{\varDelta }_{{{{{{\rm{r}}}}}}}{G}_{11}^{^\circ }+{E}_{{{{{{\rm{ads}}}}}},{{{{{\rm{H}}}}}}}\\ +T{S}_{{{{{{\rm{H}}}}}}}^{^\circ }+{\varDelta }_{{{{{{\rm{sol}}}}}},2}{G}_{{{{{{{\rm{H}}}}}}}^{\ast }}^{^\circ }+2({E}_{{{{{{\rm{ads}}}}}},{{{{{\rm{O}}}}}}}+T{S}_{{{{{{\rm{O}}}}}}}^{^\circ }+{\varDelta }_{{{{{{\rm{sol}}}}}},2}{G}_{{{{{{{\rm{O}}}}}}}^{\ast }}^{^\circ })$$

The detailed procedures to derive Eq. (4a–e) are presented in Supplementary Method. In the above equations, ${\triangle }_{{{{{{\rm{r}}}}}}}{G}_{{\it{i}}}^{^\circ }$ (*i* = 6 −11) are defined in Fig. 3b and also in the Supplementary Method. ${S}_{{{{{{\rm{HO}}}}}}}^{^\circ }$, ${S}_{{{{{{\rm{O}}}}}}}^{^\circ }$, and ${S}_{{{{{{\rm{H}}}}}}}^{^\circ }$ are standard entropies of the radicals HO^•^, O^••^, and H^•^, respectively. ${\triangle }_{{{{{{\rm{sol}}}}}},2}{G}_{{{{{{{\rm{HO}}}}}}}^{\ast }}^{^\circ }$, ${\triangle }_{{{{{{\rm{sol}}}}}},2}{G}_{{{{{{{\rm{O}}}}}}}^{\ast }}^{^\circ }$, and ${\triangle }_{{{{{{\rm{sol}}}}}},2}{G}_{{{{{{{\rm{H}}}}}}}^{\ast }}^{^\circ }$ are the changes in the standard *G* value associated with the solvation of adsorbates HO^*^, O^*^, and H^*^, respectively, from the gas phase in water (for their definition, see the Supplementary Method). All these standard thermodynamic data necessary for the calculation of ∆_r_*G*_*i*_ (*i* = 1−5) are summarized in Table 2. These results suggest that ∆_r_*G*_*i*_ (*i* = 1−5) at any given pH are functions of three variables, *E*_ads,HO_, *E*_ads,HO_, and *E*_ads,H_. Namely, the partition function *x*_1_ is the function of these three variables.

**Table 2:** Standard thermodynamic data.

Item	Value	Item	Value
${\triangle }_{{{{{{\rm{r}}}}}}}{G}_{6}^{^\circ }$	−0.86^a^	${\triangle }_{{{{{{\rm{r}}}}}}}{G}_{13}^{^\circ }$	4.92^b^
${\triangle }_{{{{{{\rm{r}}}}}}}{G}_{7}^{^\circ }$	−0.86^b^	${S}_{{{{{{\rm{HO}}}}}}}^{^\circ }$	1.9 × 10^−3c^
${\triangle }_{{{{{{\rm{r}}}}}}}{G}_{8}^{^\circ }$	0.12^b^	${S}_{{{{{{\rm{O}}}}}}}^{^\circ }$	1.7 × 10^−3c^
${\triangle }_{{{{{{\rm{r}}}}}}}{G}_{9}^{^\circ }$	1.87^b^	${S}_{{{{{{\rm{H}}}}}}}^{^\circ }$	1.2 × 10^−3c^
${\triangle }_{{{{{{\rm{r}}}}}}}{G}_{10}^{^\circ }$	2.52^b^	${\triangle }_{{{{{{\rm{sol}}}}}},2}{G}_{{{{{{\rm{HO}}}}}}\ast }^{^\circ }$	1.20^d^
${\triangle }_{{{{{{\rm{r}}}}}}}{G}_{11}^{^\circ }$	6.68^b^	${\triangle }_{{{{{{\rm{sol}}}}}},2}{G}_{{{{{{\rm{O}}}}}}\ast }^{^\circ }$	1.20^d^
${\triangle }_{{{{{{\rm{r}}}}}}}{G}_{12}^{^\circ }$	3.59^b^	${\triangle }_{{{{{{\rm{sol}}}}}},2}{G}_{{{{{{{\rm{H}}}}}}}^{\ast }}^{^\circ }\,$	0.81^d^

^a^The data were obtained by DFT calculations using the method described in the Supplementary Method section (unit: eV/particle).

^b^The data were calculated using the data of Supplementary Table 3 (unit: eV/particle).

^c^The data were directly taken from the chemistry handbook^66^ (unit: eV/particle/K).

^d^The data were estimated from the combination of experiment and computation using the method described in the Supplementary Method section (unit: eV).

To reduce the number of variables, the relationship between *E*_ads,HO_, *E*_ads,HO_, and *E*_ads,H_ was further studied. We calculated the *E*_ads,HO_, *E*_ads,O_, and *E*_ads,H_ for a series of material surfaces, which include the (100), (110), and (111) surfaces of transition metals and the (111) surfaces of the fluorites MO_2_ and the rutiles MX_2_ (for parameter settings of the calculations, see Supplementary Data 1). The results are plotted in Fig. 3c. As seen from this figure, *E*_ads,HO_ and *E*_ads,O_ have a good linear relation,

5 $${E}_{{{{{{\rm{ads}}}}}},{{{{{\rm{O}}}}}}}=1.87{E}_{{{{{{\rm{ads}}}}}},{{{{{\rm{HO}}}}}}}+1.42$$

A similar linear relation between *E*_ads,HO_ and *E*_ads,O_ has been reported previously by Nørskov^67^, Liu^68^, and coworkers, which can be ascribed to both HO^•^ and O^••^ preferring the same adsorption sites on the surfaces. Substituting ${E}_{{{{{{\rm{ads}}}}}},{{{{{\rm{O}}}}}}}$ in Eq. (4d) and Eq. (4e) by Eq. (5), plugging the values of Table 2 into Eq. (4a–e), and taking *T* = 298.15 K and pH = 7, ∆_r_*G*_*i*_ (*i* = 1−5) can be simplified as follows:

6a $${\varDelta }_{{{{{{\rm{r}}}}}}}{G}_{1}=-1.3$$

6b $${\varDelta }_{{{{{{\rm{r}}}}}}}{G}_{2}={E}_{{{{{{\rm{ads}}}}}},{{{{{\rm{HO}}}}}}}+1.4$$

6c $${\varDelta }_{{{{{{\rm{r}}}}}}}{G}_{3}={E}_{{{{{{\rm{ads}}}}}},{{{{{\rm{H}}}}}}}+2.1$$

6d $${\varDelta }_{{{{{{\rm{r}}}}}}}{G}_{4}=2.87{E}_{{{{{{\rm{ads}}}}}},{{{{{\rm{HO}}}}}}}+8.54$$

6e $${\varDelta }_{{{{{{\rm{r}}}}}}}{G}_{5}=3.74{E}_{{{{{{\rm{ads}}}}}},{{{{{\rm{HO}}}}}}}+{E}_{{{{{{\rm{ads}}}}}},{{{{{\rm{H}}}}}}}+16.4$$

Substituting ∆_r_*G*_*i*_ (*i* = 1−5) in Equation(4) with Eq. (6a–e), *x*_1_ becomes a function of only two variables, *E*_ads,HO_ and *E*_ads,H_. Figure 3d plots the variation in *x*_1_ with *E*_ads,HO_ and *E*_ads,H_. From Fig. 3d, the criterion *x*_1_ > 0.5 becomes the adsorption energy criterion of Eq. (3). Using a similar method, the adsorption energy criterion at any other pH can be obtained.

### High-throughput calculations

The high-throughput calculations of *E*_ads,H_ and *E*_ads,HO_ for the 2D materials were performed by the python script (Supplementary Software 1). The calculated adsorption energies are present in Supplementary Data 3. Briefly, the script screens the 2D materials deposited in the C2DB database that satisfy the following conditions: (1) they have considerable thermodynamic stability (∆*H*_hull_ < 0.2 eV/atom); (2) they have considerable kinetic stability (${\tilde{{{\omega }}}}_{\min }^{2}\, > \,{10}^{-5}{{{{{{\rm{eV}}}}}}/{{\AA }}}^{2}$); (3) they contain no more than two elements; (4) they have bandgap information calculation with the HSE method; (5) they have only CBM or VBM with the orbital energy located in between (−0.16 eV, 0.94 eV). A total of 126 materials were screened out from the database. To calculate *E*_ads,H_ and *E*_ads,HO_ for each of these materials, we saved the unit cell structure for the material, which contains the three-unit cell vectors (**a**, **b**, and **c**) and coordinates for all the atoms. We first checked the lengths of vectors **a** and **b**: if the length of **a** (**b**) is >1.5 times that of **b** (**a**), the length of **b** (**a**) will be doubled to build the new unit cell. We further checked the lengths of **a** and **b** for the cell: if the length of **a** or **b** is no larger than 5 Å, both **a** and **b** will be doubled. Then, all symmetrically unique adsorption sites for the cell were found to build the initial H and HO adsorption structures. All these adsorption structures were geometrically optimized using the spin-polarized DFT and crude convergence criteria, in which the total energy and force convergence were 10^−3^ eV and 0.3 eV/Å, respectively and the gamma point approximation was also used. The lowest-energy adsorption structure according to these preliminary calculations was recalculated with tighter convergence criteria: the total energy and force convergence was 10^−5^ eV and 0.02 eV/Å, respectively; a (3 × 3 × 1) k-point mesh was used. For all these calculations, the unit cell parameters were frozen. The python materials genomics (Pymatgen)^69^ and atomic simulation environment^70^ packages were used to prepare the inputs of these calculations.

### Experiments

The synthesis and characterization of the MIL-53(Fe)-X (X = NH_2_, CH_3_, H, OH, F, Cl, Br, and NO_2_) compounds have been reported in our recent study^42^. The reduction potential values of the MIL-53(Fe)-X discussed in this work were also taken from the previous study^42^. Because the values were measured using a saturated calomel electrode (SCE) and in a buffer solution with pH = 4.5, the values were converted to those with respect to HE and at pH = 7 using the following equation:

$$\varphi {{{{{\rm{HE}}}}}}({{{{{\rm{pH}}}}}}=7)=\varphi {{{{{\rm{SCE}}}}}}({{{{{\rm{pH}}}}}}=4.5)-0.0592\times \varDelta {{{{{\rm{pH}}}}}}+0.245$$

where ∆pH is the difference between the target pH and the pH of the measurement, namely ∆pH = 7 − 4.5 = 2.5.

The SOD-like activities of the MIL-53(Fe)-X (X = NH_2_, CH_3_, H, OH, F, Cl, Br, and NO_2_) MOFs were measured with the O_2_^•−^ specific fluorescent probe hydroethidine. The probe could react with O_2_^•−^, which was generated from the xanthine and xanthine oxidase, to give a fluorescent product ethidium peaked around 600 nm. More O_2_^•−^ eliminated by the MOF would give a lower fluorescent intensity, demonstrating a higher SOD-like activity of MOF. In a typical experiment, 0.6 mM xanthine, 0.05 U/mL xanthine oxidase, and 0.09 mg/mL MOFs were first added into the 0.1 M phosphate buffer (pH = 7.4), vortexed for a while, and then kept at 37 °C for 30 min. 0.1 mg/mL hydroethidine probe was then added, vortexed, and kept for another 30 min. At last, the solution was taken out and measured to assess the SOD-like activities of MOFs.

The synthesis and SOD-like activities of another MIL-47(V)-X (X = H, Br, NH_2_) MOF have been reported in our previous work^43^. Cyclic voltammograms measurements of the MIL-47(V)-X (X = H, Br, NH_2_) MOFs were carried out on an electrochemical workstation (CHI 660E) with a standard three-electrode system. 4 mg MOFs and 1 mg black were first added to the solution containing 0.48 mL of ethanol, 0.5 mL of water, and 0.02 mL of Nafion. After 30 min sonication, 0.1 mL of the mixture was dropped onto a carbon cloth and dried at room temperature. And then, the carbon cloth, an SCE, and a platinum sheet were used as the working electrode, the reference electrode, and the counter electrode, respectively.

## Supplementary information

Supplementary Information

Description of Additional Supplementary Files

Supplementary Data 1

Supplementary Data 2

Supplementary Data 3

Supplementary Software 1

## Data Availability

The authors declare that all other data supporting the findings of this study are available within the paper, the Supplementary Information/Source Data file.

## Code availability

The tailored developed codes used in this work are available within the Supplementary Software file. Density functional theory calculations are done with the code VASP (version 5.4.4).
