# Supplementary material for: Accelerated discovery of superoxide-dismutase nanozymes via high-throughput computational screening
Source: Nat Commun. 2021 Nov 25;12:6866. doi: 10.1038/s41467-021-27194-8 (PMC8616946; doi:10.1038/s41467-021-27194-8)
Supplement: Supplementary file 1 — Supplementary Information [file 41467_2021_27194_MOESM1_ESM.pdf]

Supplementary Information

Accelerated discovery of superoxide-dismutase nanozymes via high-throughput computational screening

Zhenzhen Wang, et al.

Supplementary Methods

Derivation of eqs. 4a−4e

According to the reaction paths shown in Fig. 3**a**, the following five reactions can be written for $\Delta_r G_i$ (i = 1−5),

$$O_2^{\bullet-}(aq) + H^+(aq) \longrightarrow 0.5\ H_2O_2(l) + 0.5\ O_2(g), \Delta_r G_1 \quad (S1a)$$
$$O_2^{\bullet-}(aq) + H^+(aq) + *\ (aq) \longrightarrow HO^*(aq) + 0.5\ O_2(g), \Delta_r G_2 \quad (S1b)$$
$$O_2^{\bullet-}(aq) + H^+(aq) + *\ (aq) \longrightarrow H^*(aq) + O_2(g), \Delta_r G_3 \quad (S1c)$$
$$O_2^{\bullet-}(aq) + H^+(aq) + 2*(aq) \longrightarrow HO^*(aq) + O^*(aq), \Delta_r G_4 \quad (S1d)$$
$$O_2^{\bullet-}(aq) + H^+(aq) + 3*(aq) \longrightarrow H^*(aq) + 2O^*(aq), \Delta_r G_5 \quad (S1e)$$

In reactions S1a−S1e, $\Delta_r G_i$ (i = 1−5) are Gibbs free energy (*G*) changes of the reactions; the superscript asterisks (*) designate the species to be surface adsorbates; labels aq, l, and g in parentheses designate the species to be in aqueous solution, liquid, and gas phase, respectively.

Reactions S1a−S1e can be expressed with the Hess cycles as illustrated in Fig. 3**b**. The reactions involved in this Hess cycles and the corresponding changes of *G* are defined as follow,

$$O_2^{\bullet-}(aq) + H^+(aq) \longleftrightarrow HO_2^{\bullet}(g), \Delta_r G_6^{\circ} \quad (S2)$$
$$HO_2^{\bullet}(g) \longrightarrow 0.5\ H_2O_2(l) + 0.5\ O_2(g), \Delta_r G_7^{\circ} \quad (S3a)$$
$$HO_2^{\bullet}(g) \longrightarrow HO^{\bullet}(g) + 0.5\ O_2(g), \Delta_r G_8^{\circ} \quad (S3b)$$
$$HO_2^{\bullet}(g) \longrightarrow H^{\bullet}(g) + O_2(g), \Delta_r G_9^{\circ} \quad (S3c)$$
$$HO_2^{\bullet}(g) \longrightarrow HO^{\bullet}(g) + O^{\bullet\bullet}(g), \Delta_r G_{10}^{\circ} \quad (S3d)$$
$$HO_2^{\bullet}(g) \longrightarrow H^{\bullet}(g) + 2O^{\bullet\bullet}(g), \Delta_r G_{11}^{\circ} \quad (S3e)$$
$$HO^{\bullet}(g) + *\ (g) \longrightarrow HO^*(g), \Delta_{ads} G_{HO^{\bullet}}^{\circ} \quad (S4a)$$
$$HO^*(g) \longrightarrow HO^*(aq), \Delta_{sol} G_{HO^*}^{\circ} \quad (S4b)$$
$$H^{\bullet}(g) + *\ (g) \longrightarrow H^*(g), \Delta_{ads} G_{H^{\bullet}}^{\circ} \quad (S5a)$$
$$H^*(g) \longrightarrow H^*(aq), \Delta_{sol} G_{H^*}^{\circ} \quad (S5b)$$
$$O^{\bullet\bullet}(g) + *\ (g) \longrightarrow O^*(g), \Delta_{ads} G_{O^{\bullet\bullet}}^{\circ} \quad (S6a)$$
$$O^*(g) \longrightarrow O^*(aq), \Delta_{sol} G_{O^*}^{\circ} \quad (S6b)$$
$$*\ (g) \longrightarrow *\ (aq),\ \Delta_{sol} G_*^{\circ} \quad (S7)$$

In eqs. S2 and S3a−S3e, $\Delta_r G_i^{\circ}$ (i = 6−11) are the changes of standard-state *G* for the reactions; in eqs. S4b, S5b, and S6b, $\Delta_{sol} G_{R^*}^{\circ}$ (R = HO, H, O) are the changes of *G* for the solvation of the adsorbates; in eqs. S4a, S5a, and S6a, $\Delta_{ads} G_R^{\circ}$ (R = $HO^{\bullet}$, $H^{\bullet}$, $O^{\bullet\bullet}$) are the changes of *G* for the surface adsorptions of radical R in gas phase; in eq 7, $\Delta_{sol} G_*^{\circ}$ is the change of *G* for the solvation of the nanomaterial.

Obviously, the above reactions have the follow relationships,

(S1a) = (S2) + (S3a)
(S1b) = (S2) + (S3b) + (S4a) + (S4b) – (S7)
(S1c) = (S2) + (S3c) + (S5a) + (S5b) – (S7)
(S1d) = (S2) + (S3d) + (S4a) + (S4b) + (S6a) + (S6b) – 2 × (S7)
(S1e) = (S2) + (S3e) + (S5a) + (S5b) + 2 × [(S6a) + (S6b)] – 3 × (S7)

Therefore, $\Delta_r G_i$ (i = 1−5) at the standard state (denoted as $\Delta_r G_i^{\circ}$ hereafter) can be rewritten as follow,

$$\Delta_r G_1^{\circ} = \Delta_r G_6^{\circ} + \Delta_r G_7^{\circ} \quad (S8a)$$
$$\Delta_r G_2^{\circ} = \Delta_r G_6^{\circ} + \Delta_r G_8^{\circ} + \Delta_{ads} G_{HO^{\bullet}}^{\circ} + \Delta_{sol} G_{HO^*}^{\circ} - \Delta_{sol} G_*^{\circ} \quad (S8b)$$
$$\Delta_r G_3^{\circ} = \Delta_r G_6^{\circ} + \Delta_r G_9^{\circ} + \Delta_{ads} G_{H^{\bullet}}^{\circ} + \Delta_{sol} G_{H^*}^{\circ} - \Delta_{sol} G_*^{\circ} \quad (S8c)$$
$$\Delta_r G_4^{\circ} = \Delta_r G_6^{\circ} + \Delta_r G_{10}^{\circ} + \Delta_{ads} G_{HO^{\bullet}}^{\circ} + \Delta_{sol} G_{HO^*}^{\circ} + \Delta_{ads} G_{O^{\bullet\bullet}}^{\circ} + \Delta_{sol} G_{O^*}^{\circ} - 2\Delta_{sol} G_*^{\circ} \quad (S8d)$$

$$\Delta_r G_5^\circ = \Delta_r G_6^\circ + \Delta_r G_{11}^\circ + \Delta_{ads} G_{H^\bullet}^\circ + \Delta_{sol} G_{H^*}^\circ + 2(\Delta_{ads} G_{O^{\bullet\bullet}}^\circ + \Delta_{sol} G_{O^*}^\circ) - 3\Delta_{sol} G_*^\circ \quad \text{(S8e)}$$

Rewriting $\Delta_{ads} G_R^\circ$ as follow,

$$\Delta_{ads} G_R^\circ = \Delta_{ads} H_R^\circ - T \times \Delta_{ads} S_R^\circ \quad \text{(S9a)}$$

where $\Delta_{ads} H_R^\circ$ and $\Delta_{ads} S_R^\circ$ are the changes of standard-state enthalpy (H) and entropy (S) for the surface adsorption of radical R in gas phase. Because $\Delta_{ads} H_R^\circ$ is mainly contributed by the change of total energy ($E_{ads,R}$) and $\Delta_{ads} S_R^\circ$ the reduction of the radical's S to zero, eq S9a can be reformulated as

$$\Delta_{ads} G_R^\circ = E_{ads,R} + TS_R^\circ \quad \text{(S9b)}$$

We define the following relationships:

$$\Delta_{sol,2} G_{HO^*}^\circ = \Delta_{sol} G_{HO^*}^\circ - \Delta_{sol} G_*^\circ \quad \text{(S9c)}$$

$$\Delta_{sol,2} G_{H^*}^\circ = \Delta_{sol} G_{H^*}^\circ - \Delta_{sol} G_*^\circ \quad \text{(S9d)}$$

$$\Delta_{sol,2} G_{O^*}^\circ = \Delta_{sol} G_{O^*}^\circ - \Delta_{sol} G_*^\circ \quad \text{(S9e)}$$

Substituting eqs. S9b-S9e into eqs. S8a–S8e, $\Delta_r G_i^\circ$ (i = 1–5) can be formulated as follow,

$$\Delta_r G_1^\circ = \Delta_r G_6^\circ + \Delta_r G_7^\circ \quad \text{(S10a)}$$

$$\Delta_r G_2^\circ = \Delta_r G_6^\circ + \Delta_r G_8^\circ + E_{ads,HO} + TS_{HO}^\circ + \Delta_{sol,2} G_{HO^*}^\circ \quad \text{(S10b)}$$

$$\Delta_r G_3^\circ = \Delta_r G_6^\circ + \Delta_r G_9^\circ + E_{ads,H} + TS_H^\circ + \Delta_{sol,2} G_{H^*}^\circ \quad \text{(S10c)}$$

$$\Delta_r G_4^\circ = \Delta_r G_6^\circ + \Delta_r G_{10}^\circ + E_{ads,HO} + TS_{HO}^\circ + \Delta_{sol,2} G_{HO^*}^\circ + E_{ads,O} + TS_O^\circ + \Delta_{sol,2} G_{O^*}^\circ \quad \text{(S10d)}$$

$$\Delta_r G_5^\circ = \Delta_r G_6^\circ + \Delta_r G_{11}^\circ + E_{ads,H} + TS_H^\circ + \Delta_{sol,2} G_{H^*}^\circ + 2(E_{ads,O} + TS_O^\circ + \Delta_{sol,2} G_{O^*}^\circ) \quad \text{(S10e)}$$

Then, $\Delta_r G_i$ (i = 1–5) at any pH can be obtained by,

$$\Delta_r G_i = \Delta_r G_i^\circ + 0.0592\text{pH} \quad \text{(S11)}$$

Substituting eqs. S9a–S9e into eq. S10, eqs. 4a–4e can be obtained.

Calculation of $\boldsymbol{\Delta_r G_6^\circ}$

The value of $\Delta_r G_6^\circ$ was calculated based on the following reactions:

$$O_2^{\bullet-}(aq) + H_3O^+(aq) \longleftrightarrow HO_2^\bullet(g) + H_2O(aq), \Delta_r G_6^\circ \quad \text{(S12a)}$$

$$\Delta_r G_6^\circ = G_{HO_2^\bullet}^\circ(g) + G_{H_2O}^\circ(aq) - G_{O_2^{\bullet-}}^\circ(aq) - G_{H_3O^+}^\circ(aq) \quad \text{(S12b)}$$

In eq. S12b, $G_{HO_2^\bullet}^\circ(g)$ is the standard G of $HO_2^\bullet$ in gas phase; $G_{H_2O}^\circ(aq)$, $G_{O_2^{\bullet-}}^\circ(aq)$, and $G_{H_3O^+}^\circ(aq)$ are the standard G for H_2O, $O_2^{\bullet-}$, and H_3O^+ in water, respectively. These four values were obtained by frequency analysis calculations based on the optimized molecular geometries. The B3LYP method(*1, 2*) in conjugation with the 6-311++G(d,p) basis set (*3, 4*) as implemented in the Gaussian 09 program (*5*) was used for these calculation. The solvation effect of water was considered using the SMD model.

Calculation of $\boldsymbol{\Delta_r G_i^\circ}$ ***(i = 7–11)***

The values of $\Delta_r G_i^\circ$ (i = 7–11) were calculated using the thermodynamic data taken from the chemistry handbook(*6*). The thermodynamic data that were used to calculate the $\Delta_r G_i^\circ$ (i = 7–11) was shown in Supplementary Table 2. According to eqs. S1a–S1e,

$$\begin{aligned}\Delta_r G_7^\circ &= 0.5\,\Delta_f G_m^\circ\,(H_2O_2) + 0.5\,\Delta_f G_m^\circ\,(O_2) - \Delta_f G_m^\circ\,(HO_2^\bullet)\\ &= 0.5 \times (-120.4) + 0.5 \times 0 - 22.6\\ &= -82.8\ (\text{kJ mol}^{-1})\\ &= -0.86\ (\text{eV})\end{aligned}$$

$$\Delta_r G_8^\circ = \Delta_f G_m^\circ\,(HO^\bullet) + 0.5\,\Delta_f G_m^\circ\,(O_2) - \Delta_f G_m^\circ\,(HO_2^\bullet)$$
$$= 34.2 + 0.5 \times 0 - 22.6$$
$$= 11.6\ (\mathrm{kJ\ mol^{-1}})$$
$$= 0.12\ (\mathrm{eV})$$
$$\Delta_r G_9^\circ = \Delta_f G_m^\circ\,(H^\bullet) + \Delta_f G_m^\circ\,(O_2) - \Delta_f G_m^\circ\,(HO_2^\bullet)$$
$$= 203.3 + 0 - 22.6$$
$$= 180.7\ (\mathrm{kJ\ mol^{-1}})$$
$$= 1.87\ (\mathrm{eV})$$
$$\Delta_r G_{10}^\circ = \Delta_f G_m^\circ\,(HO^\bullet) + \Delta_f G_m^\circ\,(O^{\bullet\bullet}) - \Delta_f G_m^\circ\,(HO_2^\bullet)$$
$$= 34.2 + 231.7 - 22.6$$
$$= 243.3\ (\mathrm{kJ\ mol^{-1}})$$
$$= 2.52\ (\mathrm{eV})$$
$$\Delta_r G_{11}^\circ = \Delta_f G_m^\circ\,(H^\bullet) + 2 \times \Delta_f G_m^\circ\,(O^{\bullet\bullet}) - \Delta_f G_m^\circ\,(HO_2^\bullet)$$
$$= 203.3 + 2 \times 231.7 - 22.6$$
$$= 644.1\ (\mathrm{kJ\ mol^{-1}})$$
$$= 6.68\ (\mathrm{eV})$$

Estimation of $\Delta_{sol,2}G_{HO^*}^\circ$, $\Delta_{sol,2}G_{O^*}^\circ$, and $\Delta_{sol,2}G_{H^*}^\circ$

$\Delta_{sol,2}G_{R^*}^\circ$ are defined in eqs. S9c-S9e. Such solvation energies are hard to determine either experimentally or computationally. For the purpose of estimation, the values of $\Delta_{sol,2}G_{HO^*}^\circ$ and $\Delta_{sol,2}G_{O^*}^\circ$ were supposed to be equal and their values on difference material surfaces were also supposed to be equal. Then, the values of $\Delta_{sol,2}G_{HO^*}^\circ$ and $\Delta_{sol,2}G_{H^*}^\circ$ could be estimated by the combination of Fig. 3**d** and available experimental results. According to the experimental results of Table 1, noble metal Pd and Pt had the SOD-like activities while defect-free CeO_2 did not have the activity. Such information can be used to determine the border of the line corresponding to $x_1 = 0.5$ in Fig. 3**d**, which in turn could be used to derive the values of $\Delta_{sol,2}G_{HO^*}^\circ$ and $\Delta_{sol,2}G_{H^*}^\circ$. Using this method, the values of $\Delta_{sol,2}G_{HO^*}^\circ$, $\Delta_{sol,2}G_{O^*}^\circ$, and $\Delta_{sol,2}G_{H^*}^\circ$ were estimated to be 1.2 eV, 1.2 eV, and 0.81 eV, respectively.

Calculation of electronic density of states

The geometry optimization of all nano-surfaces of Supplementary Fig.2 were performed using the projector augmented wave (PAW) method(*7*) implemented in the Vienna Ab initio Simulation Package (VASP)(*8*). The Perdew–Burke–Ernzerhof (PBE)(*9*) exchange-correlation functional with the generalized gradient approach (GGA) was applied. The cutoff energy of plane-wave was 400 eV. The vacuum values of 15 Å along *z* orientation were set to avoid interaction between units. The electronic and geometry optimization convergence criteria were set to 10^{-5} eV and 0.02 eV $Å^{-1}$, respectively. For metallic nano-surfaces, such as Au(111), Ag(111), Pt(111), Pt(111), graphene, Nb_2C and V_2C, gaussian smearing with a width of 0.2 eV was used for the Fermi level. However, for semiconductive nano-surfaces, such as NiO(100), MoS_{2-x}, Mn_3O_4(001), Co_3O_4(001), δ-MnO_2, MnO(001) and CeO_2(111), gaussian smearing with a width of 0.05 eV was used for the Fermi level. Electronic density of state (DOS) of all nano-surfaces were calculated in the level of Heyd-Scuseria-Ernzerhof (HSE06)(*10*) hybrid functional using the optimized structure in PBE + U GGA function. More calculated parameters, such as thickness and supercell size of slabs, *K*-points and U_{eff} values, one could see Supplementary Table 1.

Calculation of adsorption energies

Because only the most thermodynamically favorable adsorption sites are relevant, the adsorption energies of metals, metal oxides, and metal fluorides were calculated only for the sites that are most likely to have the strongest affinities for the corresponding adsorbates. The surface adsorption chemistry of these materials have already been well studied before. According to the known knowledge, the hydroxyl (HO·) group slightly prefers to adsorb on the "hollow" or "bridge" sites on metal surfaces, where the oxygen can coordinate with more metals(*11*). So does the hydrogen atom(*11*). This might be understood by that both hydroxyl and hydrogen adsorbates accept electrons from the surrounding metals, which subsequently have electrostatic attractions with the surrounding metals. So, both adsorbates prefer to form the closely-packed configurations with their surrounding metals to maximize the nonmetal-metal coordination numbers. As for metal oxides and fluorides, the hydroxyl group prefers to adsorb on the surface metals because these metals have less coordination numbers than metals in the bulk and are coordinatively unsaturated. In contrast, the hydrogen atom prefers to adsorb on the O or F sites on metal oxides and fluorides, because atomic hydrogen is highly reductive and prefers sites with strong electron affinity (e.g., atoms with strong electronegativity). For these reasons, the hydroxyl and hydrogen adsorption energies for all metal surfaces were calculated based on the computationally relaxed adsorption configurations where the adsorbates were located near the hollow sites of the surfaces. The hydroxyl adsorption energies for all metal oxides and fluorides were calculated based on the configurations where the hydroxyls were near the surface metals and the hydrogen adsorption energies were calculated based on the configurations where the hydrogen atoms were near the O or F sites in the surfaces. All these relaxed adsorption configurations could be readily obtained by geometry optimizations not using symmetry constraints and using the initial structures where the groups were placed near the desired sites.

MIL-53(Fe)-H contains many possible addition sites for hydroxyl and hydrogen radicals. However, only several are probable for the side reactions illustrated in Fig. 3**a**. In the MIL-53(Fe)-H structure, each iron is coordinated with five oxygen atoms; it has a free d-orbital for further covalent bonding. Therefore, irons in the MIL-53(Fe)-H structure can well model irons in the surfaces of the MOF materials. Except for irons, all the other atoms in the MIL-53(Fe)-H structure are covalently saturated. So, irons are the only probable sites for the addition of hydroxyls on MIL-53(Fe)-H. Atomic hydrogen is a highly reductive racial, which prefers to adsorb on atoms with strong electronegativity. So, oxygen atoms are the most probable sites for the additions of hydroxyls on MIL-53(Fe)-H. Although the additions of hydroxyls and hydrogens to other sites of MIL-53(Fe)-H are possible to yield products with even larger thermodynamic stabilities, these additions severely destructed the structure of MIL-53(Fe)-H and did not need to be considered because they were unlikely the side reactions of $O_2^{\cdot -}$. The one-dimensional nanowire structure was used to model the MOF structure (e.g., see Supplementary Fig.6). Supplementary Fig. 7 comparably shows the adsorption energies of H on different sites. As can be seen from Supplementary Fig.7, oxygen bridging two irons is indeed the most favorable site for hydrogen.

Energies vs. hydrogen electrode (HE) potential

The energy levels of a material surface with respect to HE potential ($E_{\text{vs HE}}$) were calculated using the following procedures:

1) Calculating the electronic band structure for the material using the slab model, which obtains energy bands ($E_{\text{vs fermi}}$) with respect to Fermi energy (E_{fermi}) for the material;

2) Calculating vacuum energy (E_{vac}) for the NM;

3) Calculating $E_{vs\ HE}$ using this equation,

$$E_{vs\ HE} = E_{vac} - E_{vs\ fermi} - E_{fermi} - 4.5$$

in which 4.5 eV is the scaling factor relating the normal hydrogen electrode scale (NHE) to absolute vacuum scale (AVS);

Characterization of ceria particles

Diffuse reflectance ultraviolet-visible (UV-vis) spectra were recorded using an Agilent Cary 5000 ultraviolet-visible-infrared spectrometer ($BaSO_4$ as a reference). Valence-band X-ray photoelectron spectra were given by an ESCALab220i-XL spectrometer equipped with a twin-anode Al K_α (1486.6 eV) X-ray source.

Calculation of optical energy band-gap for ceria particles

The optical band-gap (E_g) of samples was determined according to the following equation,

$$(\alpha h\nu)^n = A(h\nu - E_g)$$

where hν is the photon energy (*J*), *A* is a proportionality constant, and *n* is an index that indicates the nature of transition (*n* = 1/2 for indirect transition; *n* = 2 for direct transition). Considering that CeO_2 nanoparticles are claimed to be an indirect band-gap material (*n* = 2), the recorded UV-vis spectra were first transformed to the curve of $(\alpha h\nu)^2$ versus $h\nu$ and the variations of $(\alpha h\nu)^2$ versus $h\nu$ were plotted. The straight line range of these plots was extrapolated to the *x*-axis (y = 0) to obtain the values of E_g.

Calculation of the energy levels for ceria particles

These energy levels (versus standard hydrogen electrode, SHE) of conduction band (E_{CB}, V) and valence band (E_{VB}, V) were calculated using the following empirical equations:

$$E_{CB} = X + 4.5 - 0.5E_g$$

$$E_{VB} = E_{CB} - E_g$$

where *X* is given using the following equation,

$$X = [\chi(\mathrm{Ce})\chi(O)^2]^{\frac{1}{3}}$$

χ is the electronegativity of samples and is given from the following Equation,

$$\chi = \frac{\mathrm{I} + A}{2}$$

where *I* (eV) and *A* (eV) are the ionization energy and electron affinity of element Ce (5.54 eV and 0.52 eV) and O (13.62 eV and 1.46 eV) (*6*), respectively.

Finally, the Fermi level is given by the following equation,

$$E_F = E_{VB} - \Delta E_{VB}$$

where ΔE_{VB} (V) is the energy of the valence band with respect to the Fermi level.

Supplementary Note 1. Work function (W_f) version of the energy level principle for metals.

For metal surfaces, the Fermi energies with respect to the vacuum energy are known as W_f. So, the W_f-version of energy level principle can be obtained:

$$\varphi_1 + 4.5 < W_f < \varphi_2 + 4.5$$

In the above equation, 4.5 V is the difference between vacuum energy and the hydrogen potential. The W_f of the elemental substances available in the Lange's Handbook of Chemistry(*6*) are plotted in Supplementary Fig. 3**b**. As can be seen, Au, Pt, and Pd have their W_f located in the range. Because W_f is usually measured in an ultrahigh vacuum condition, which is quite different from the aqueous condition for the substances to function as SOD mimics. Unlike noble metals, which are chemically inert, many substances of Supplementary Fig. 3**b** easily react with O_2 or water at room temperature, which would dramatically change their W_f. So, the result of Supplementary Fig. 3**b** cannot always be directly used to predict whether the elemental substances are potential SOD mimics.

Supplementary Note 2. Verification of the energy level principle with ceria particles.

Recent experimental study has demonstrated that ceria particles synthesized under the same condition except with at different temperatures, 0, 30, 60, and 90 ℃ (denoted as Ceria_0, Ceria_30, Ceria_60, and Ceria_90, hereafter) have markedly different SOD-like activities: Ceria_0 and Ceria_30 have much stronger SOD-like activities than Ceria_60 and Ceria_90 [see Fig. 3A of Ref. (*12*)]. To check whether these ceria particles obey the energy level principle, the frontier molecular orbitals of these ceria samples were measured by the combination of experiments and calculations. To this end, the UV−vis diffuse reflectance spectra (DRS) of these ceria particles were measured (Supplementary Fig. 8). These DRS spectra were then transformed to the $(\alpha h\nu)^2$ versus $h\nu$ plots to obtain the gap between valence band and conduction band and the gap between valence band and defect levels (Supplementary Fig. 9). The energies from the valence bands to the Fermi levels were measured using the XPS valence band spectra (Supplementary Fig. 10). Because the energy levels (versus hydrogen electrode, HE) of conduction band (E_{CB}, V) and valence band (E_{VB}, V) of ceria particles cannot be measured using the ultraviolet photoelectron spectroscopy because of poor electron conductivity of the samples, these energies were calculated using the method described in the above Supplementary Method section. The results are shown in Supplementary Fig. 11. As can been seen, ceria_0 and ceria_30 rather than ceria-60 or ceria_90 have the iFMO, which agrees with their SOD-like activity order and verifies the energy level principle.

Supplementary Note 3. Computational support for the predicted SOD-like activities of the 2D materials.

To our knowledge, 14 of the 121 2D materials have been experimentally realized. These 14 materials include nine transition metal dichalcogenide (TMDC) structures. All these nine TMDCs have been chosen for computational investigation to check their SOD-like activities. The CBM and VBM energies of these nine TMDC structures are shown in Supplementary Table 3. As can be seen from this table, six structures including ZrS_2, PtS_2, $ZrSe_2$, ZnS_2, HfS_2, and VS_2 have their CBMs located in the range (−0.16 eV, 0.94 eV). According to our energy-energy principle, they should follow the LUMO-mediated mechanism to mimic the activity of SOD. Similarly, the remaining three structures including $MoTe_2$, WSe_2, and WTe_2 have their VBMs in the range and thus they should follow the HOMO-mediated mechanism. To verify this prediction, we have located the intermediates and transition-state structures involved in both the HOMO- and LUMO-mediated pathways for ZrS_2 and $MoTe_2$ and plotted the reaction energy profiles in Supplementary Fig. 12. As shown in Supplementary Fig. 12**a**, the energy barrier of the rate-determining step (RDS) of the LUMO-path is much lower than that of the RDS of the HOMO-path (0.83 eV *vs* 2.38 eV), suggesting that ZrS_2 indeed prefers the LUMO-mediated mechanism, in good agreement with the prediction. On the other hand, Supplementary Fig. 12**b** suggests that $MoTe_2$ prefers the HOMO-mediated mechanism, which also agrees with the prediction. For the remaining seven TMDC structures, their kinetically favorable pathways were investigated by locating only the intermediate structures. As shown in Supplementary Fig. 13, the LUMO-mediated paths for HfS_2, SnS_2, PtS_2, $ZrSe_2$, and VS_2 do not contain any intermediate structures with relatively high energies, in agreement with the prediction that these structures prefer the LUMO-mediated mechanism. Similarly, the results of Supplementary Fig. 14 suggest that the HOMO-mediated mechanisms for feasible for WTe_2 and WSe_2, in agreement with the prediction. Therefore, these computational results have supported the SOD-like activities predicted by the high throughput calculations.

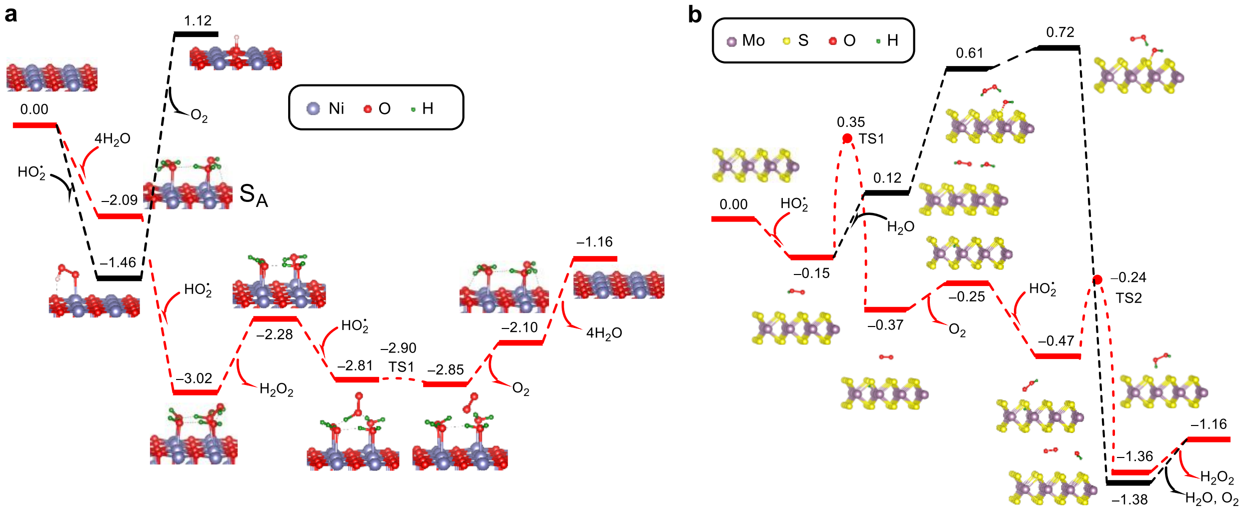

Supplementary Fig. 1. Reaction energy profiles indicating the different mechanisms of the NMs. a The surface of NiO(100) prefers the mechanism (red) in which the surface is first oxidized (forming the H_2O_2) and then reduced back (forming the O_2); the mechanism (black) is kinetically disfavored. **b** The surface of MoS_{2-x} prefers the mechanism (red) while the mechanism (black) is less kinetically competitive. In A, structure labeled with S_A was used as the starting point for the reaction energy profile of Fig. 2**f**.

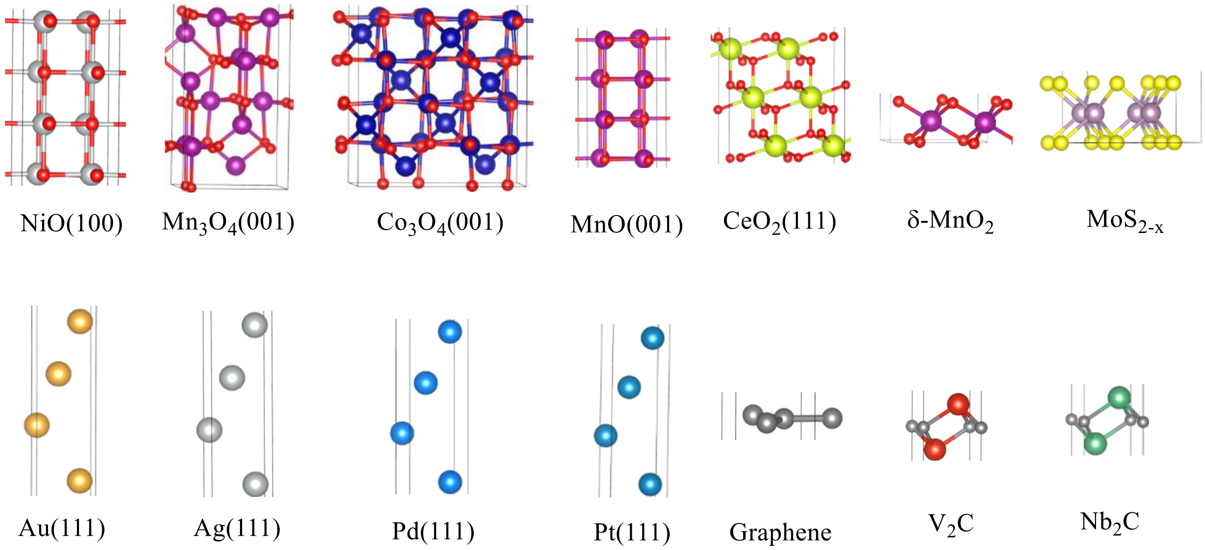

Supplementary Fig. 2. Slab models used for the calculations of electronic density of states. The details of these calculations can be found in the Supplementary Methods.

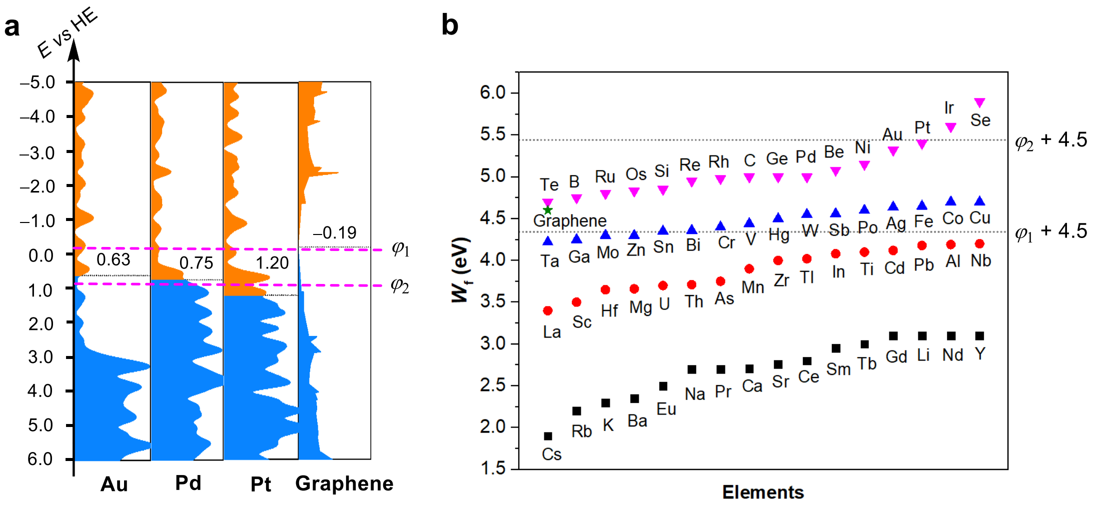

Supplementary Fig. 3. The energy level principle for NMs to catalytically scavenge $O_2^{\bullet-}$. a Calculated electronic density of states with energies of Fermi energies marked. **b** Work functions (W_f) of the elemental substances; the values for metals are taken from Lange's Handbook of Chemistry(*6*) and that for graphene is from Ref. (*13*).

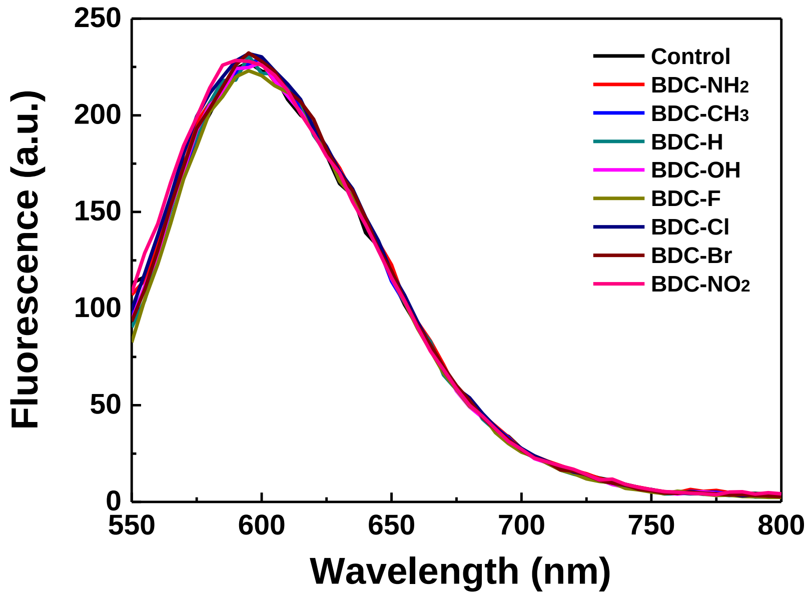

Supplementary Fig. 4. Fluorescent spectra for monitoring the SOD-like activities of the ligands BDC-X. BDC stands for 1,4-benzenedicarboxylic acid, X = NH_2, CH_3, H, OH, F, Cl, Br, and NO_2.

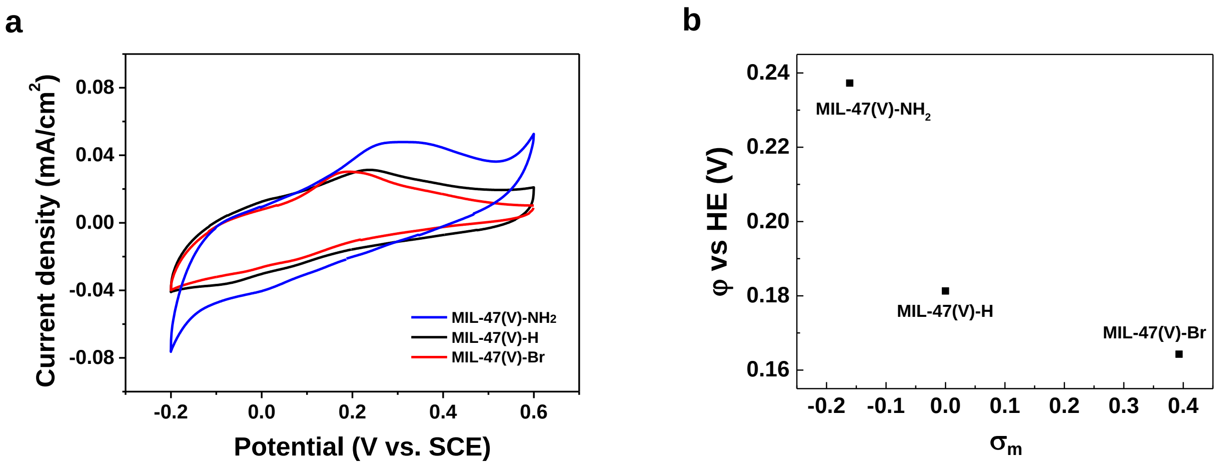

Supplementary Fig. 5. Cyclic voltammograms and reduction potentials of MIL-47(V)-X MOFs (X = H, NH_2, Br). a Cyclic voltammograms of MIL-47(V)-X with respect to SCE. **b** The reduction potentials of MIL-47(V)-X with respect to hydrogen electrode (HE) at pH = 7.

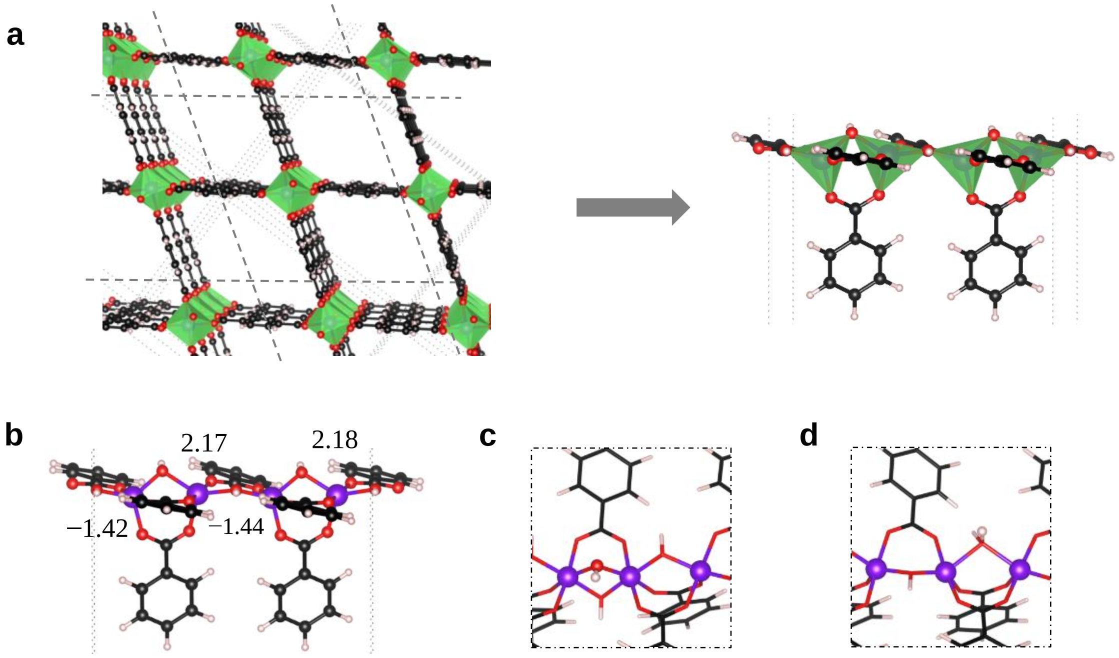

Supplementary Fig. 6. Theoretical calculations of MIL-47(V)-H. a Building the one-dimensional line model for MIL-47(V)-H from the crystal bulk structure. **b** Calculated structure of the MIL-47(V)-H. The magnetic moments (in μB) of irons are labelled. **c** The adsorbed $HO^{\cdot}$ structure of MIL-47(V)-H. **d** The adsorbed $H^{\cdot}$ structure of MIL-47(V)-H. V, purple; H, white; O, red; C, black.

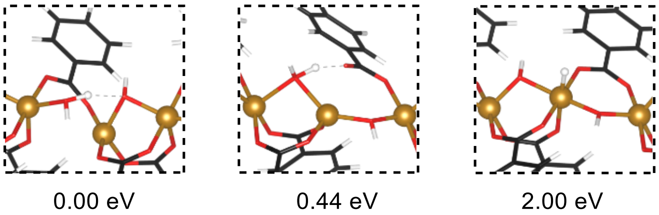

Supplementary Fig. 7. Energies for the adsorptions a hydrogen atom at different sites of MIL-53(Fe)-H. In the left panel, the H atom was adsorbed on the oxygen bridging two irons and made a hydrogen bond with the neighboring bridging oxygen. In the middle panel, the H atom was also adsorbed on the oxygen bridging two irons but made a hydrogen bond with the ligand oxygen. In the right panel, the H atom was adsorbed on the iron. In all these panels, the iron and adsorbed hydrogen atoms were shown in the ball-and-stick mode and all the other atoms in the stick mode. Relative energies of these adsorption configurations were marked.

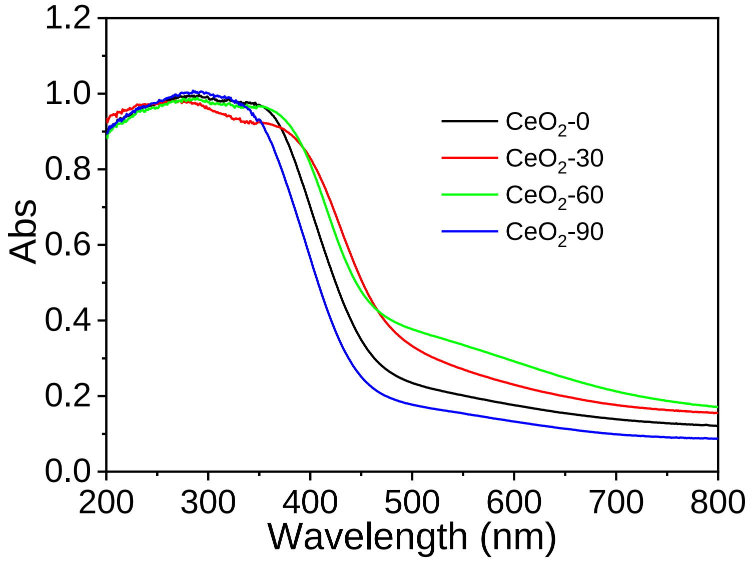

Supplementary Fig. 8. UV−vis diffuse reflectance spectra (DRS) of CeO_2_0, CeO_2_30, CeO_2_60, and CeO_2_90. These DRS spectra were used to obtain the gap between valence band and conduction band and the gap between valence band and defect levels for the samples, and the details can be found in the Supplementary Notes.

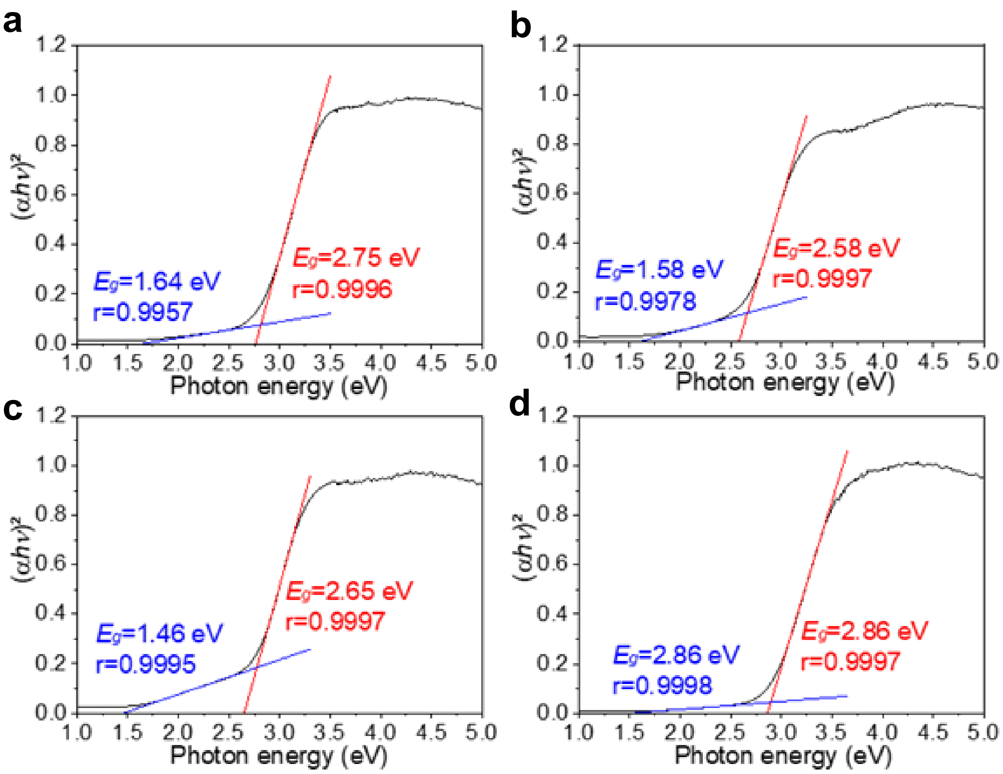

Supplementary Fig. 9. $(\alpha h\nu)^2$ versus *hν* plot. a CeO_2_0. **b** CeO_2_30. **c** CeO_2_60. **d** CeO_2_90. This shows the gap between valence band and conduction band (red), as well as the gap between valence band and defect levels (blue).

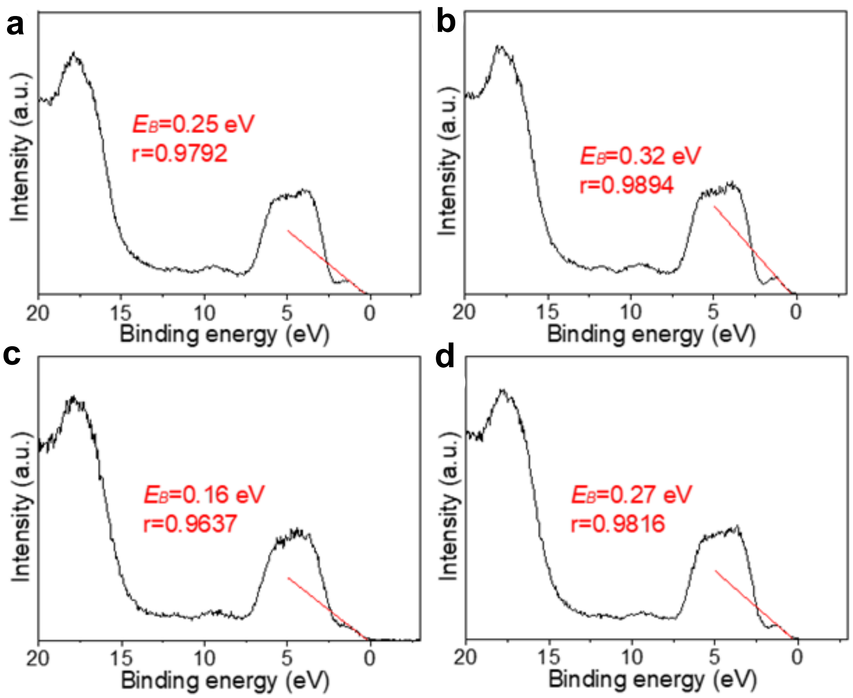

Supplementary Fig. 10. XPS valence band spectra. **a** CeO_2_0. **b** CeO_2_30. **c** CeO_2_60. **d** CeO_2_90. E_B means the energy from valence band to Fermi levels.

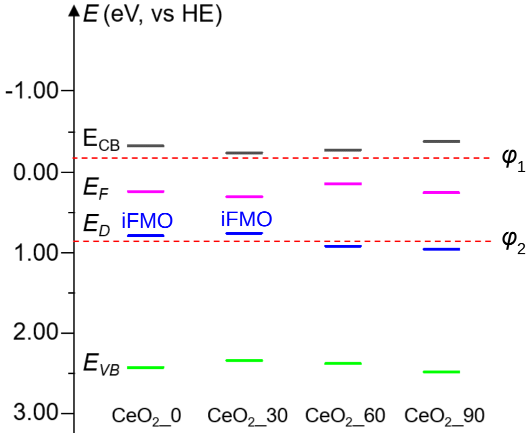

Supplementary Fig. 11. Experimental verification using ceria particles synthesized under the same condition except with at different temperature, 0, 30, 60, and 90 °C. Energies of conduction bands (E_{CB}), Fermi levels (E_F), defect levels (E_D), and valance bands (E_{VB}) of the ceria particles are shown in the figure. Ceria_0 and Ceria_30 have the intermediate frontier molecular orbital (iFMO), in agreement with their higher SOD-like activisties reported in Ref. (12).

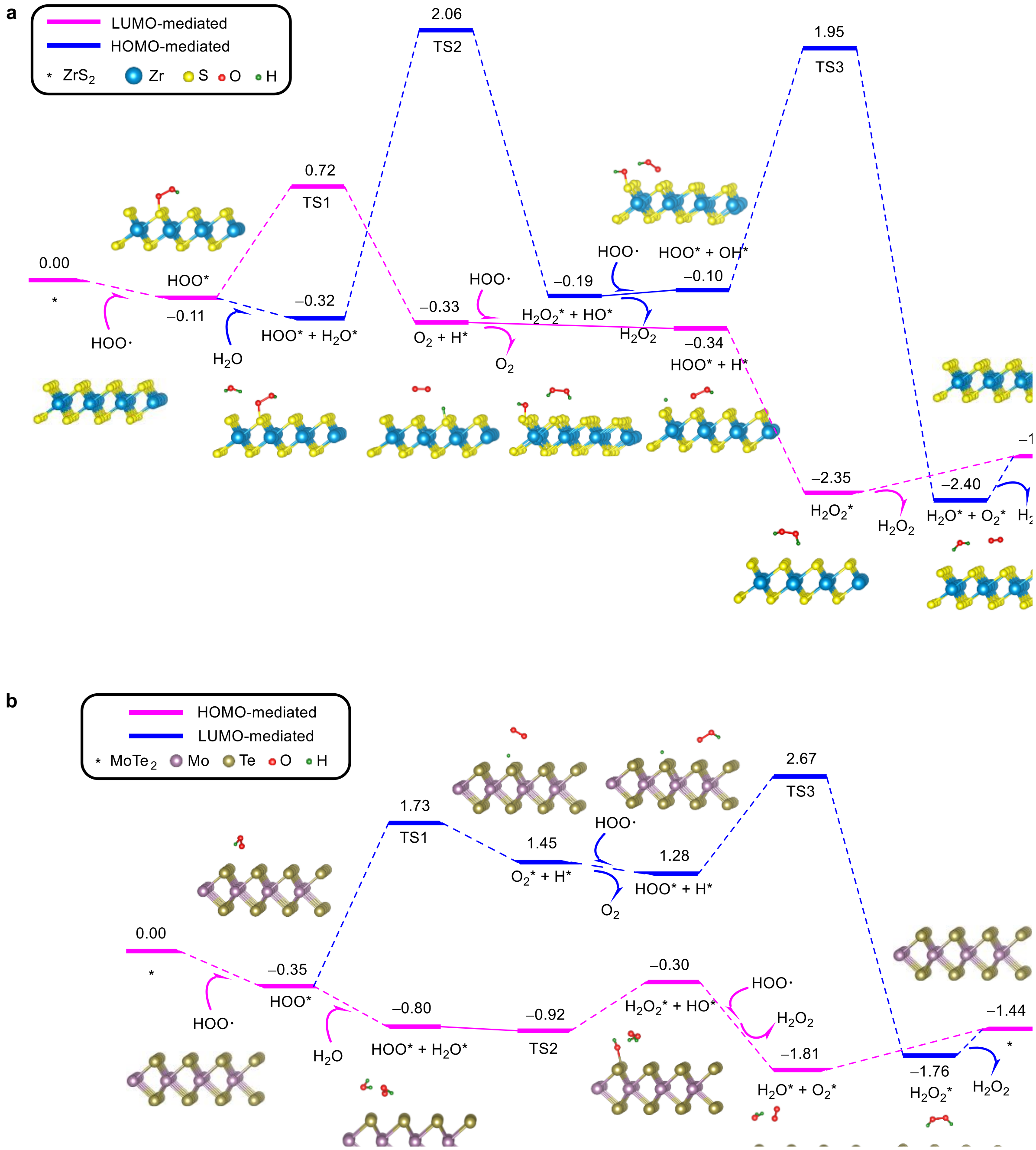

Supplementary Fig. 12. Comparison of HOMO- and LUMO-mediated pathways for the SOD-like catalysis. a ZrS_2. **b** $MoTe_2$. In **a**, the LUMO-mediated pathway is more kinetically favorable than the HOMO-mediated pathway; in **b**, the HOMO-mediated pathway is instead more kinetically favorable.

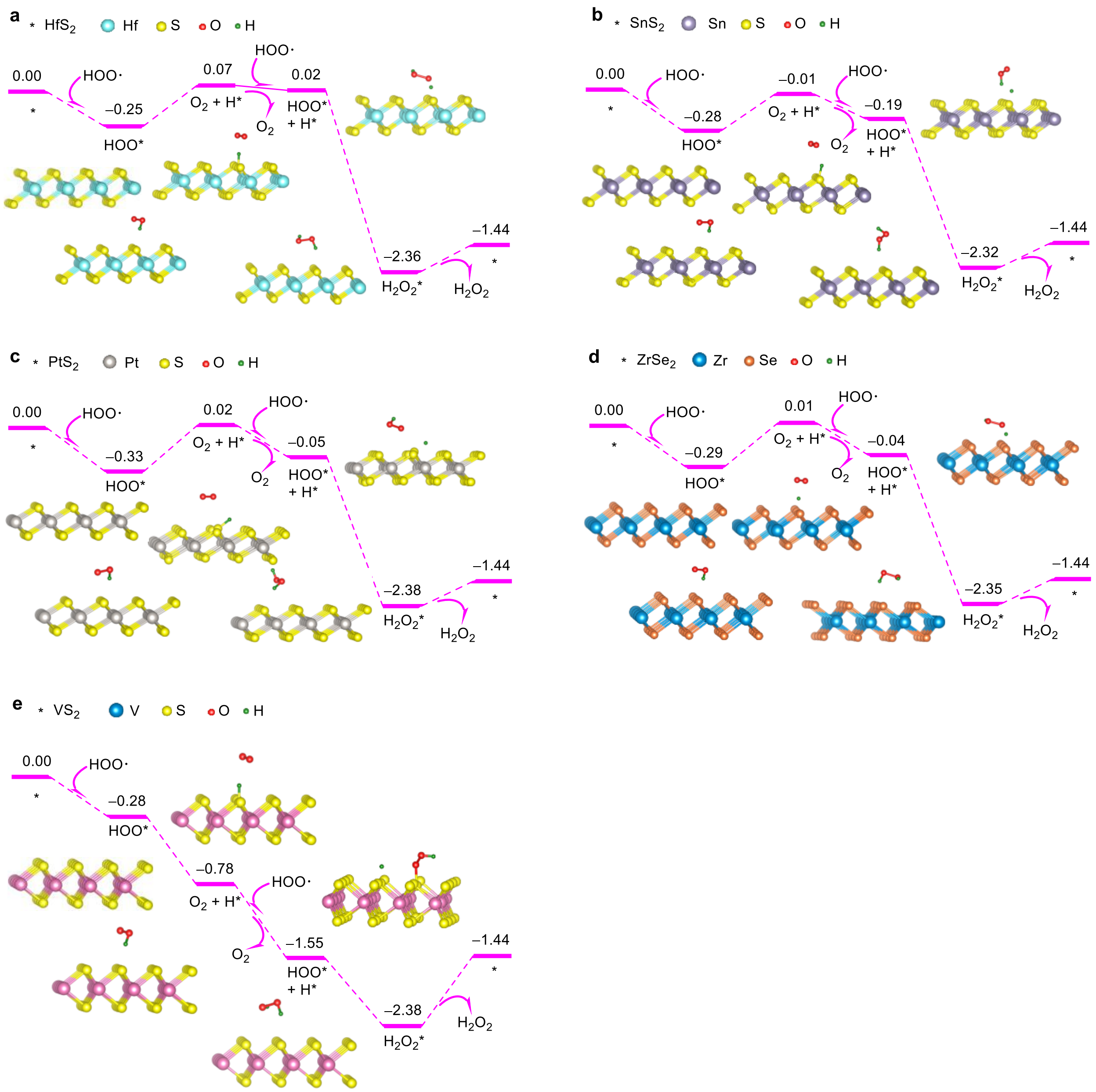

Supplementary Fig. 13. LUMO-mediated SOD-like catalysis of TMDC structures. a HfS_2. **b** SnS_2. **c** PtS_2. **d** $ZrSe_2$. **e** VS_2. The energy profiles suggested to the feasibility of the LUMO-mediated pathway for these materials.

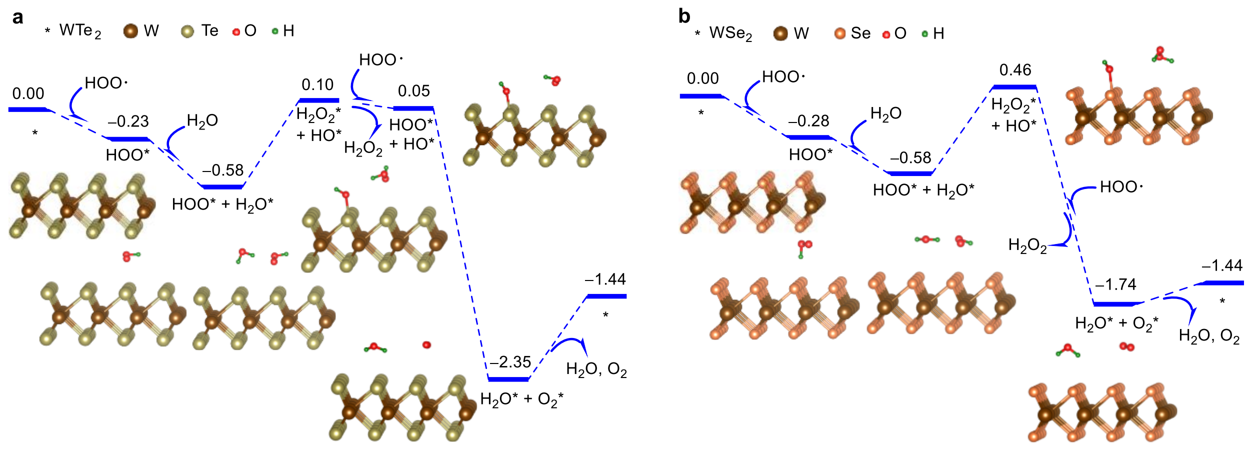

Supplementary Fig. 14. HOMO-mediated SOD-like catalysis of TMDC structures. a WTe_2. **b** WSe_2. The energy profiles suggested to the feasibility of the HOMO-mediated pathway for these materials.

Supplementary Table 1. Calculating parameters for the nano-surfaces.

Slabs	Thickness (layers)	Supercell	*K*-points	U_{eff} for metals (eV)	Magnetism
NiO(100)	4	1 × 1	7 × 7 × 1	5.3(*14*, *15*)	AFM
MoS_{2-x}	3	4 × 4	11 × 11 × 1	4(*16*)	NM
Mn_3O_4(001)	9	1 × 1	7 × 7 × 1	4(*17*)	AFM
Co_3O_4(001)	9	1 × 1	5 × 5 × 1	3.5(*18*)	FM
δ-MnO_2	3	1 × 1	13 × 15 × 1	4(*19*)	FM
MnO(001)	4	1 × 1	5 × 5 × 1	4(*20*)	AFM
CeO_2(111)	9	1 × 2	5 × 5 × 1	5(*21*, *22*)	NM
Au(111)	4	1 × 1	15 × 15 × 1		NM
Ag(111)	4	1 × 1	17 × 17 × 1		NM
Pt(111)	4	1 × 1	15 × 15 × 1		NM
Pd(111)	4	1 × 1	15 × 15 × 1		NM
Graphene	1	1 × 1	15 × 15 × 1		NM
V_2C	3	1 × 1	11 × 11 × 1		NM
Nb_2C	3	1 × 1	11 × 11 × 1		NM

Supplementary Table 2. Energies of Frontier Molecular Orbitals of the TMDC Structures.

No.	material	$E_{VBM\ vs\ Vac}$ (eV)	$E_{CBM\ vs\ Vac}$ (eV)	$E_{VBM\ vs\ HE}$ (eV)	$E_{CBM\ vs\ HE}$ (eV)
1	ZrS_2	−6.97	−4.80	2.47	0.30
2	PtS_2	−6.87	−4.39	2.37	−0.11
3	$ZrSe_2$	−5.89	−4.68	1.39	0.18
4	SnS_2	−7.38	−5.02	2.88	0.52
5	HfS_2	−6.89	−4.74	2.39	0.24
6	VS_2	−5.94	−5.11	1.44	0.61
7	$MoTe_2$	−4.81	−3.44	0.31	−1.06
8	WSe_2	−4.99	−3.26	0.49	−1.24
9	WTe_2	−4.50	−3.36	0.00	−1.14

Supplementary Table 3. Standard thermodynamic properties of chemical substances.

Species	$\Delta_f G_m^\circ$ (kJ/mol)
H_2O_2 (l)	−120.4[a]
H_2O (l)	−237.14[a]
O_2 (g)	0[a]
$HO^{\bullet}$ (g)	34.2[a]
$HO_2^{\bullet}$ (g)	22.6[a]
$O^{\bullet\bullet}$ (g)	231.7[a]
$H^{\bullet}$ (g)	203.3[a]

[a]The data was taken from Lange's Handbook of Chemistry(*6*).

Supplementary References.

1. K. Kim, K. D. Jordan, Comparison of density functional and MP2 calculations on the water monomer and dimer. *J. Phys. Chem.* **98**, 10089-10094 (1994).
2. P. J. Stephens, F. J. Devlin, C. F. Chabalowski, M. J. Frisch, Ab Initio calculation of vibrational absorption and circular dichroism spectra using density functional force fields. *J. Phys. Chem.* **98**, 11623-11627 (1994).
3. R. Krishnan, J. S. Binkley, R. Seeger, J. A. Pople, Self-consistent molecular orbital methods. XX. A basis set for correlated wave functions. *J. Chem. Phys.* **72**, 650-654 (1980).
4. A. D. McLean, G. S. Chandler, Contracted Gaussian basis sets for molecular calculations. I. Second row atoms, Z=11–18. *J. Chem. Phys.* **72**, 5639-5648 (1980).
5. M. J. Frisch. Gaussian 09, Revision D.01 (Gaussian, Inc., Wallingford, CT, 2009).
6. J. A. Dean, J. A. Dean, Ed. Lange's Handbook of Chemistry (McGRAW-HILL, INC., New York, 1998).
7. P. E. Blochl, Projector augmented-wave method. *Phys. Rev. B* **50**, 17953-17979 (1994).
8. G. Kresse, J. Furthmüller, Efficiency of ab-initio total energy calculations for metals and semiconductors using a plane-wave basis set. *Comp. Mater. Sci.* **6**, 15-50 (1996).
9. J. P. Perdew, K. Burke, M. Ernzerhof, Generalized gradient approximation made simple. *Phys. Rev. Lett.* **77**, 3865-3868 (1996).
10. A. V. Krukau, O. A. Vydrov, A. F. Izmaylov, G. E. Scuseria, Influence of the exchange screening parameter on the performance of screened hybrid functionals. *J. Chem. Phys.* **125**, 224106 (2006).
11. A. A. Phatak, W. N. Delgass, F. H. Ribeiro, W. F. Schneider, Density Functional Theory Comparison of Water Dissociation Steps on Cu, Au, Ni, Pd, and Pt. *J. Phys. Chem. C* **113**, 7269-7276 (2009).
12. X. Liu, J. Wu, Q. Liu, A. Lin, S. Li, Y. Zhang, Q. Wang, T. Li, X. An, Z. Zhou, M. Yang, H. Wei, Synthesis-temperature-regulated multi-enzyme-mimicking activities of ceria nanozymes. *J. Mater. Chem. B* **9**, 7238-7245 (2021).
13. T. Takahashi, H. Tokailin, T. Sagawa, Angle-resolved ultraviolet photoelectron spectroscopy of the unoccupied band structure of graphite. *Phys. Rev. B* **32**, 8317-8324 (1985).
14. K. Sebbari, J. Roques, C. Domain, E. Simoni, Uranyl ion interaction at the water/NiO(100) interface: a predictive investigation by first-principles molecular dynamic simulations. *J. Chem. Phys.* **137**, 164701 (2012).
15. F. Cinquini, L. Giordano, G. Pacchioni, A. M. Ferrari, C. Pisani, C. Roetti, Electronic structure of NiO/Ag(100) thin films from DFT+U and hybrid functional DFT approaches. *Phys. Rev. B* **74**, 165403 (2006).
16. B. Zhu, J. Lang, Y. H. Hu, S-Vacancy induced indirect-to-direct band gap transition in multilayer MoS_2. *Phys. Chem. Chem. Phys.* **22**, 26005-26014 (2020).
17. C. Franchini, R. Podloucky, J. Paier, M. Marsman, G. Kresse, Ground-state properties of multivalent manganese oxides: Density functional and hybrid density functional calculations. *Phys. Rev. B* **75**, 195128 (2007).
18. A. H. Hashim, A. O. H. Zayed, S. M. Zain, V. S. Lee, S. M. Said, Electronic, magnetic and structural properties of Co_3O_4 (100) surface: a DFT+U study. *Appl. Surf. Sci.* **427**, 1090-1095 (2018).
19. P. Wuamprakhon, A. Krittayavathananon, S. Kosasang, N. Ma, T. Maihom, J. Limtrakul, N. Chanlec, P. Kidkhunthod, M. Sawangphruk, Effect of Intercalants inside Birnessite-Type Manganese Oxide Nanosheets for Sensor Applications. *Inorg. Chem.* **59**, 15595-15605 (2020).
20. R. Nelson, P. M. Konze, R. Dronskowski, First-principles chemical bonding study of manganese carbodiimide, MnNCN, as compared to manganese oxide, MnO. *J. Phys. Chem. A* **121**, 7778-7786 (2017).
21. M. Nolan, S. Grigoleit, D. C. Sayle, S. C. Parker, G. W. Watson, Density functional theory studies of the structure and electronic structure of pure and defective low index surfaces of ceria. *Surf. Sci.* **576**, 217-229 (2005).
22. Z. X. Yang, Q. G. Wang, S. Y. Wei, D. W. Ma, Q. A. Sun, The effect of environment on the reaction of water on the ceria(111) surface: A dft plus u study. *J. Phys. Chem. C* **114**, 14891-14899 (2010).
