# Supplementary material for: Accelerated discovery of superoxide-dismutase nanozymes via high-throughput computational screening
Source: Nat Commun. 2021 Nov 25;12:6866. doi: 10.1038/s41467-021-27194-8 (PMC8616946; doi:10.1038/s41467-021-27194-8)
Supplement: Supplementary file 2 — Description of Additional Supplementary Files [file 41467_2021_27194_MOESM2_ESM.pdf]

Description of Additional Supplementary Files

File Name: Supplementary Data 1
Description: Model and parameter settings for calculating the adsorption energies of the materials surfaces.

File Name: Supplementary Data 2
Description: Source data of the figures.

File Name: Supplementary Data 3
Description: Adsorption energies for the two dimensional materials.

File Name: Supplementary Software 1
Description: A python code for the high-throughput calculation of adsorption energies.
